# Effect of acupuncture on neuroinflammatory responses in depression animals: a systematic review and meta-analysis

**DOI:** 10.3389/fpsyt.2025.1624648

**Published:** 2025-10-31

**Authors:** Dongyi Ni, Jinbu Zhang, Ruyu Qi, Yingjie Huang, Min Li, Lining Duan

**Affiliations:** ^1^ Clinical Medical of Acupuncture, Moxibustion and Rehabilitation, Guangzhou University of Chinese Medicine, Guangzhou, China; ^2^ The First Affiliated Hospital of Guangzhou University of Chinese Medicine, Guangzhou, China; ^3^ The First Clinical Medical School, Guangzhou University of Chinese Medicine, Guangzhou, China

**Keywords:** acupuncture, neuroinflammatory, depression, animals, depressive-like behaviors, meta-analysis, systematic review

## Abstract

**Background:**

Depression causes many negative effects and even death to patients; the burden on society is also heavy in terms of economics. The neuroinflammatory mechanism of acupuncture regulating depression is unclear. Thus, this study evaluated the effect of acupuncture on neuroinflammatory responses in depression animals and provided a clinical reference for depression treatment.

**Methods:**

The Web of Science, Embase, the Cochrane Library, PubMed, CNKI, WanFang, CBM, and VIP databases were searched. The SYRCLE Risk of Bias Tool was used to assess bias in the studies. Meta-analysis was performed using Stata 15.0. The sources of heterogeneity were explored using subgroup analysis, and stability was evaluated using sensitivity analysis. Quality of evidence for outcomes was assessed using GDT.

**Results:**

This study included 25 studies and 466 animals. According to meta-analysis, compared with the control group, acupuncture significantly reduced IL-1β (SMD: −1.62, 95% CI: −1.93, −1.31), IL-6 (SMD: −1.89, 95% CI: −2.51, −1.26), and TNF-α in depression animals (SMD: −2.09, 95% CI: −2.83, −1.34) and improved IL-4 (SMD: 1.01, 95% CI:0.35, 1.67), IL-10 (SMD:0.77, 95% CI:0.26, 1.28), body weight (SMD:1.69, 95% CI: 1.23, 2.15), crossing numbers (SMD: 1.74, 95% CI: 1.31, 2.17), and rearing numbers (SMD: 1.77, 95% CI: 1.16, 2.39).

**Conclusion:**

Acupuncture has the potential to alleviate depression by attenuating neuroinflammatory responses, and the mechanism may be related to modulating the release of inflammatory factors as well as regulating the activation of microglia.

**Systematic review registration:**

https://www.crd.york.ac.uk/prospero/, identifier CRD42024534237.

## 1 Introduction

Depression is a common mental health disorder characterized by a persistently low mood, loss of interest, and reduced ability to carry out daily activities. According to data from the Global Health Data Exchange Platform, 34% of adolescents aged 10–19 worldwide are at risk of developing clinical depression, exceeding the prevalence among those aged 18–25 (1, 2). As a mental illness, depression not only causes negative impacts on patients, including death, but also imposes a significant economic burden on society due to its associated clinical symptoms and medical expenses (3).

Treatment for depression typically involves both psychological and pharmacological approaches. Psychological therapy helps patients learn to cope with stress, change negative thought patterns, and modify unhealthy behavioral habits. Pharmacological treatment typically involves antidepressant medications, such as selective serotonin reuptake inhibitors (SSRIs) or tricyclic antidepressants (TCAs), as well as other pharmacological treatments (4). However, these medications require long-term use and may cause side effects such as suicidal thoughts, sexual dysfunction, and metabolic syndrome (5, 6). Therefore, finding safe and natural treatment options for depression is of great importance.

The current mainstream view on the pathogenesis of depression suggests that low levels of neurotransmitters such as 5-HT and norepinephrine, dysfunction of the hypothalamic–pituitary–adrenal axis, impaired immune function, and low levels of endocrine hormones play a key role in the development of depression. In terms of immune function, pro-inflammatory factors (IL-6, IFNs, IL-1β, TNF-α, IFNs, etc.) and anti-inflammatory factors (IL-10, IL-8, etc.) regulate the activation of microglia, leading to the sustained release of a series of inflammatory mediators that affect neurons and neurotransmitters, thereby inducing depressive symptoms in the body (7, 8). This chronic neuroinflammation mechanism is the primary mechanism underlying the development of depression. Chronic stress is a major trigger for the onset of depression and an effective method for preparing animal models of depression. Chronic stress can lead to elevated inflammatory cytokines and can cause depression through multiple pathways, including promoting oxidative stress, inducing neurotoxic effects, and inhibiting neurogenesis (9). Additionally, both clinical studies and animal models of depression have observed immune system activation. Persistently activated inflammatory cytokines can directly affect hippocampal function in the brain and indirectly influence depressive behavior and emotional regulation (10, 11). These findings suggest that inflammatory responses are closely associated with depressive disorders and are present throughout the entire process of disease onset, progression, and outcome (12, 13). However, treatment methods and drugs targeting this key point remain relatively scarce at present.

Acupuncture, as a safe, natural, and traditional treatment method, has therapeutic effects on depression (14), but its specific mechanisms of action remain unclear. It has been demonstrated that acupuncture can effectively improve depressive symptoms such as low mood and suicidal tendencies by regulating inflammatory mediators. Studies (15, 16) have shown that acupuncture reduces the levels of pro-inflammatory factors in the serum of depressed patients. Animal studies (17) have also found that acupuncture can downregulate pro-inflammatory factor levels in the prefrontal cortex of chronically stressed depressed rats and upregulate anti-inflammatory factor levels to alleviate brain inflammation. However, the specific regulatory mechanisms of these studies remain unclear, and there is a lack of systematic reviews of the evidence. Therefore, this study was conducted to evaluate the effects of acupuncture on neuroinflammatory responses in depressed animals, providing a reference for future treatments of depression.

## 2 Methods

This study has been registered with PROSPERO (CRD42024534237) and also follows systematic reviews and meta-analysis according to the latest PRISMA guidelines (18).

### 2.1 Search strategy

A combination of subject terms and entry terms were mainly used in the search, and the subject terms were mainly “depression,” “animals,” “acupuncture,” “electroacupuncture”, “MDD (Major depressive disorder)”, “CUMS (Chronic unpredictable mild stress)” and “CRS (Chronic restraint stress)”. Search was limited to August 2025. The language was limited to English and Chinese. The detailed search strategy is presented in **Supplementary Table 1**.

### 2.2 Inclusion criteria

The retrieved studies were screened independently by two authors for inclusion and exclusion. Here are the criteria for inclusion (1): depression animals (2); the test group included manual acupuncture (MA) or electroacupuncture (EA) (3); the control group included depression animals without any interventions (4); the outcomes included at least one inflammatory indicator (IL-1β, IL-6, TNF-α, IL-4, IL-10).

### 2.3 Exclusion criteria

(1) Depression combined with other diseases (2); other acupuncture or other therapies were used in the test group (3); review or clinical trial (4); non-Chinese and English studies.

### 2.4 Studies screening and data extraction

To resolve disputes, the study was reviewed by two independent researchers, the data were extracted, and a third party cross-checked it. We excluded duplicates first and then read the title after excluding studies that were obviously irrelevant, a further reading of the abstract followed, and then the full text was read in order to determine if it was included. Authors of the original studies were contacted by e-mail in order to obtain information that had not yet been identified but was crucial to the study. We extracted data on (1) first author’s name and year of publication (2), basic characteristics of depression animals (3), depression modeling methodology (4), intervention characteristics (5), outcomes (the primary outcome is inflammation, and the secondary outcomes are body weight and behaviors). If only graphical data were provided, GetData Graph Digitizer 2.26 was used to digitize the graphs.

### 2.5 Risk of bias

We assessed the risk of bias using 10 items of the SYRCLE Risk of Bias Tool, which were rated as “low risk,” “unclear,” and “high risk” (19). Differences in assessments were resolved through discussion between two evaluators, and a third investigator could be consulted if needed for judgment.

### 2.6 Statistics

Statistical analysis was performed with Stata 15.0. All outcomes variables were continuous. Since the units are not consistent, we used the standardized mean difference (SMD) and 95% confidence intervals (CI) as the effect indicator, and the difference between the data was statistically significant when P < 0.05. The heterogeneity was determined by I²; if I² ≥ 50%, the heterogeneity was more obvious and the data were analyzed using the random effects model; and if I² < 50%, the heterogeneity was less and the fixed effects model was used to analyze the data. To identify heterogeneity sources, a subgroup analysis was conducted based on different acupuncture methods and modeling in the test group and the random effects model was used to test the differences in effect size between subgroups. Sensitivity analysis was performed using the “one-by-one method” to ensure the robustness of the results. The publication bias was checked using funnel plots and Egger’s test when the number of studies exceeded 10.

### 2.7 Certainty assessment

In accordance with the grading guidelines, the evidence quality for the primary outcome was assessed using the GDT software. Several factors were taken into account, including study design domain, risk of bias, inconsistency, indirectness, imprecision, and other considerations such as publication bias, effect size, and potential confounders. Based on the evidence quality, four categories were defined: high, moderate, low, and very low.

## 3 Results

### 3.1 Literature search process

**Figure 1:**
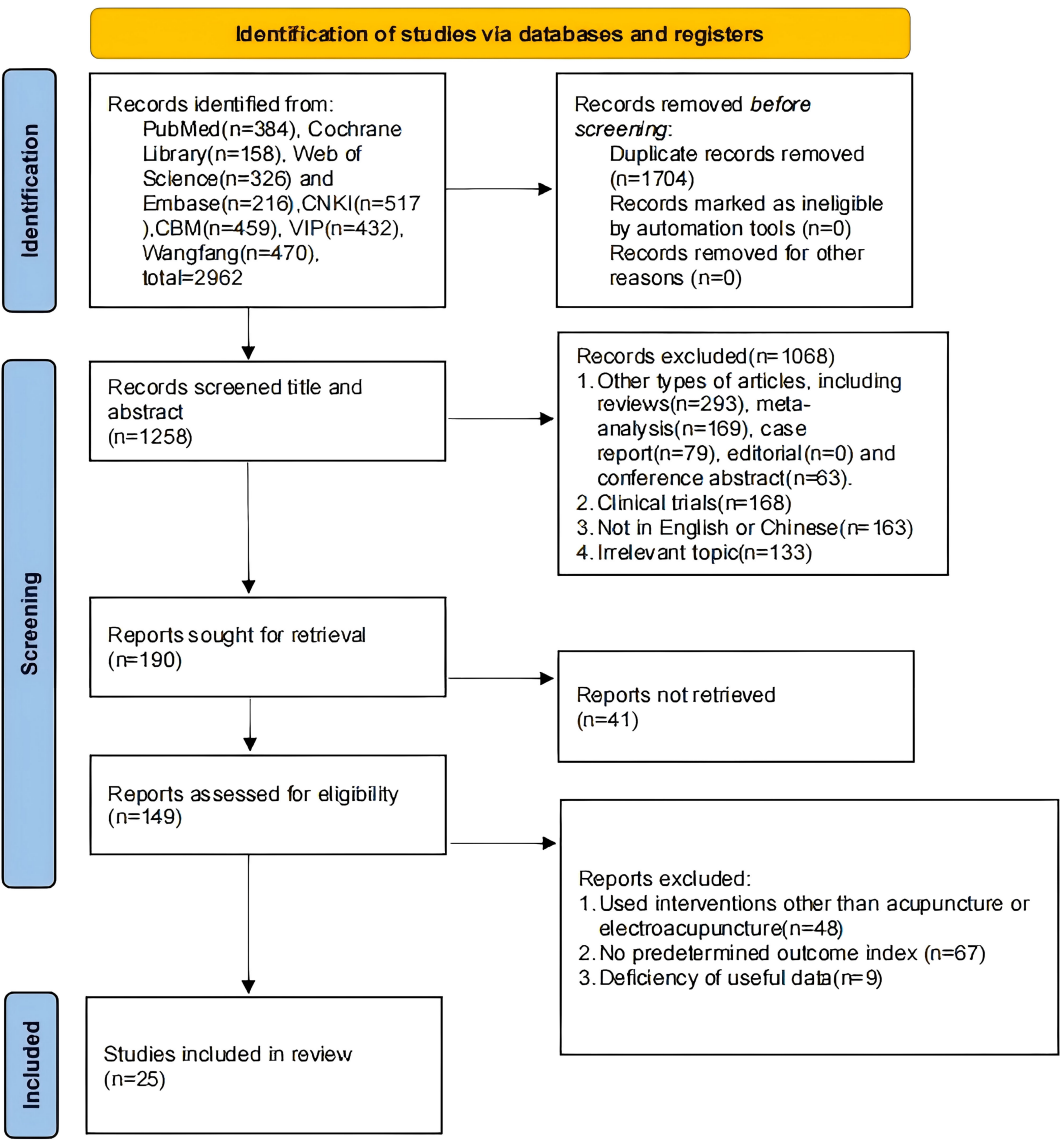
Literature search process.

We retrieved 2,962 studies, of which 1,704 duplicates were removed after import into Endnote, and 1,068 studies were eliminated by reading the titles and abstracts, of which 604 were other types of studies, 168 were clinical trials, 163 were non-Chinese and English, and 133 were irrelevant topics. Then, 41 studies were excluded without retrieving the report. By reading the full text, 48 studies using interventions other than MA or EA were excluded, 67 studies without predetermined outcomes, and 9 studies lacking useful data. A total of 25 studies were finally included (17, 20–43) (**Figure 1**).

### 3.2 Basic characteristics of the studies

A total of 25 studies and 466 animals were included in this studies, with 233 animals in each of the control and test groups (**Supplementary Table 2**).

**Figure 2:**
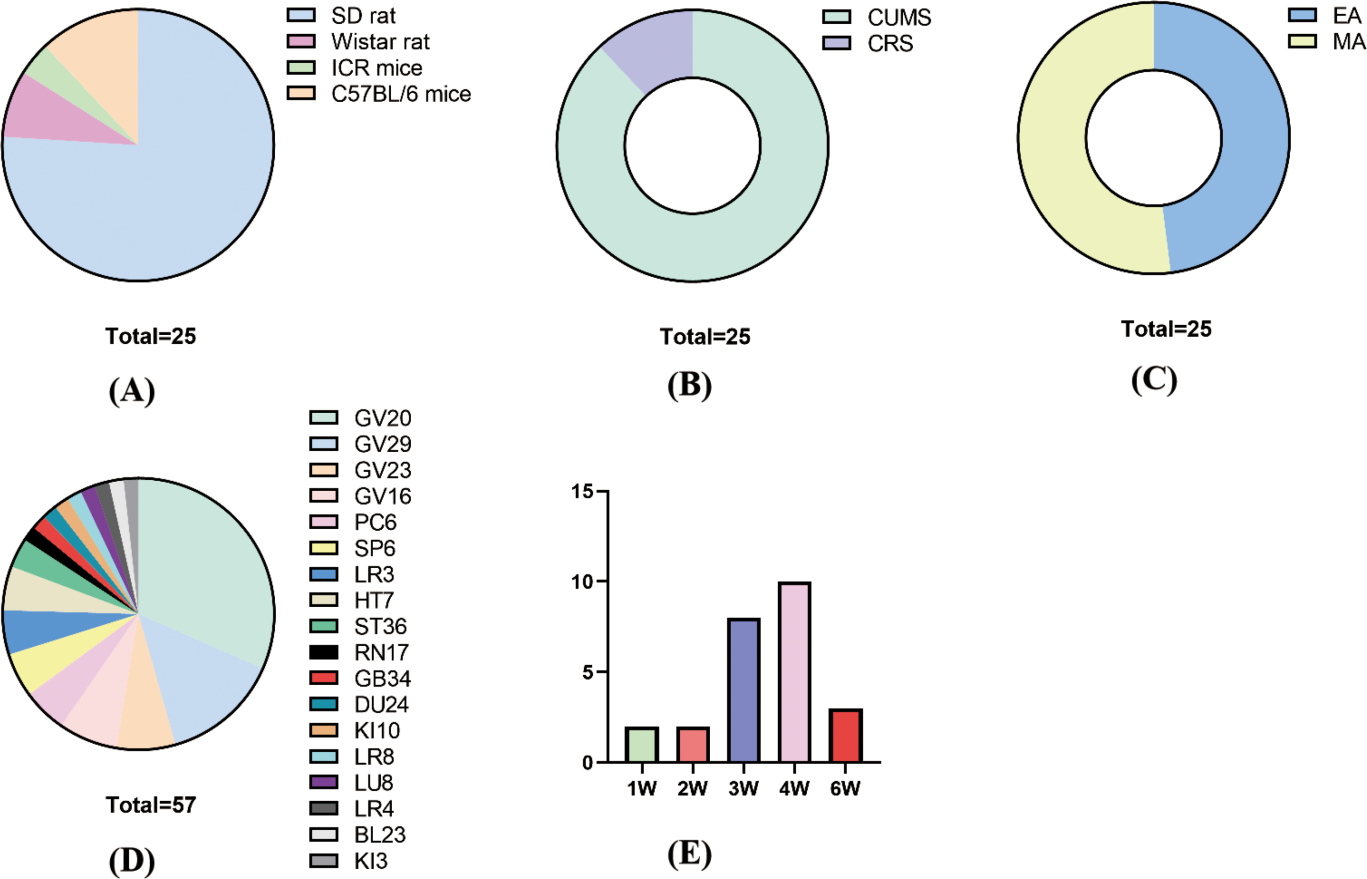
Basic characteristics. **(A)** Animal species; **(B)** molding methods; **(C)** acupuncture methods; **(D)** acupuncture points; **(F)** treatment time and course.

In terms of species and modeling methods, there were four species of rats: 19 SD rats, 3 C57BL/6 mice, 2 Wistar rats, and 1 ICR mouse (**Figure 2A**). There were 2 modeling methods: 22 rats used CUMS and 3 used CRS (**Figure 2B**).

For acupuncture methods, 12 studies used EA and 13 studies used MA (**Figure 2C**). For acupuncture points, the frequency of acupoint use, from largest to smallest, was GV20 (18) GV29 (8) GV23 (4) GV16 (4) PC6 (3) HT7 (3) SP6 (3) LR3 (3) ST36 (2), and the rest of the acupuncture points were used once. In the combination of acupoints, GV20+GC29 and GV23+GV26 were more frequently used (**Figure 2D**).

For treatment time and course, each treatment time was 10–30 min, with 30 min as the main duration. The course of treatment was 1–6 weeks, with 4 weeks as the main duration (**Figure 2E**).

For parameters of EA, the frequency used for EA was mainly 2 Hz, and the wave pattern was mainly sparse-dense wave.

Inflammatory indicators (IL-1β, IL-6, TNF-α, IL-4, and IL-10) were used as main outcomes; as secondary outcomes, body weight and open field test.

### 3.3 Risk of bias

**Figure 3:**
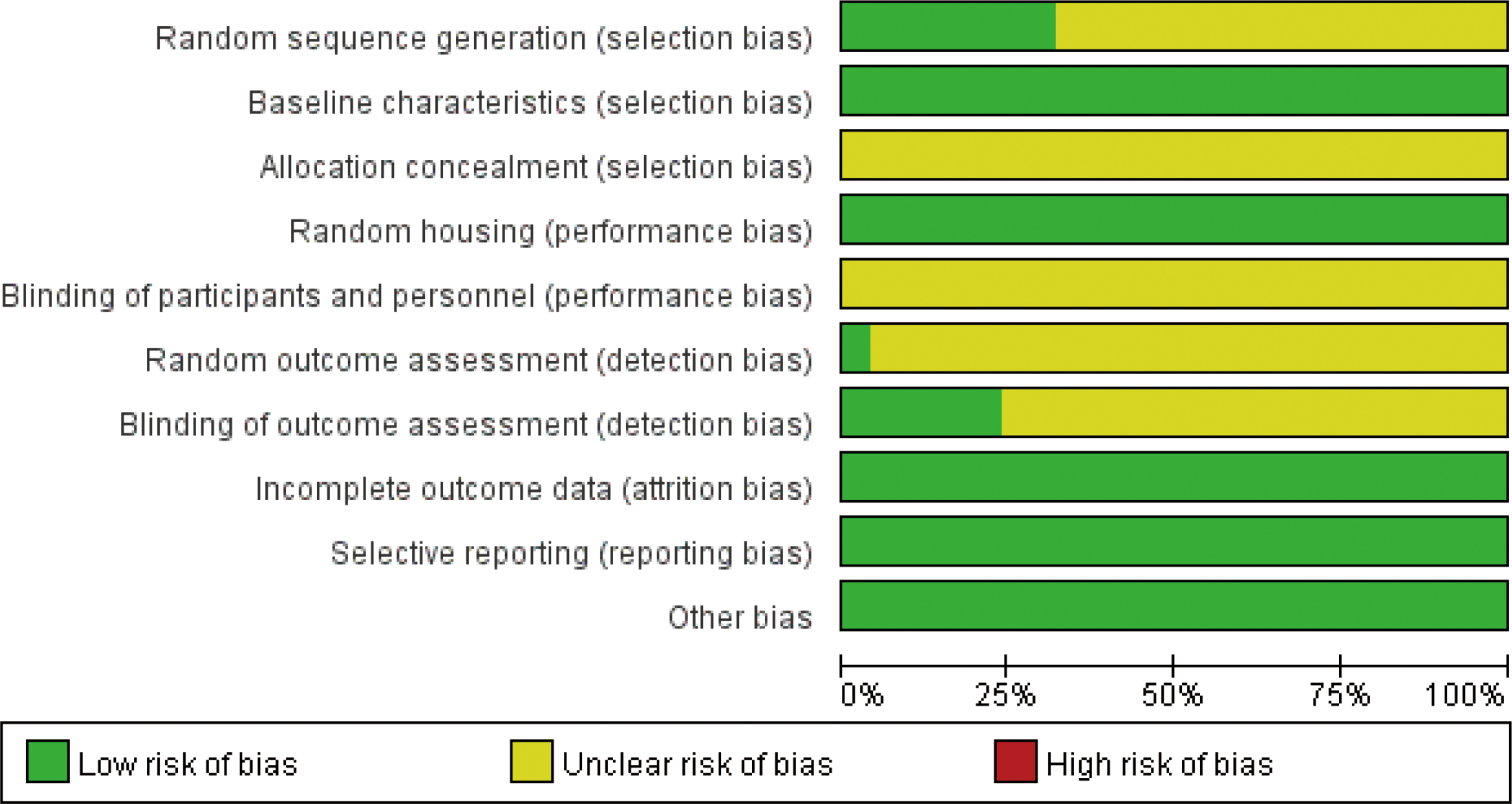
Risk of bias.

All studies were RCTs; eight studies (22, 25, 27, 29, 30, 35, 40, 41) used the method of random number table, and in the remaining studies, there was no explicit description of the random assignment method. All studies found no significant differences between baseline characteristics of the animals; therefore, they were considered low risk. All studies were referred to the manner in which the animals were allocated concealment. All studies were low risk as there were no significant differences in the animal breeding conditions. Due to the specificity of the intervention, the test group could not use well-implemented blinding. In the randomized outcome assessment, one study (22) used the lottery method for outcome assessment. Six studies (17, 22, 23, 27, 30, 36) used blinding when evaluating the outcome, and the rest were explicitly mentioned. All studies reported complete results without selective reporting, and other sources of bias were not found (**Figure 3**, **Supplementary Figure 1**).

### 3.4 Meta-analysis

#### 3.4.1 IL-1β

**Figure 4:**
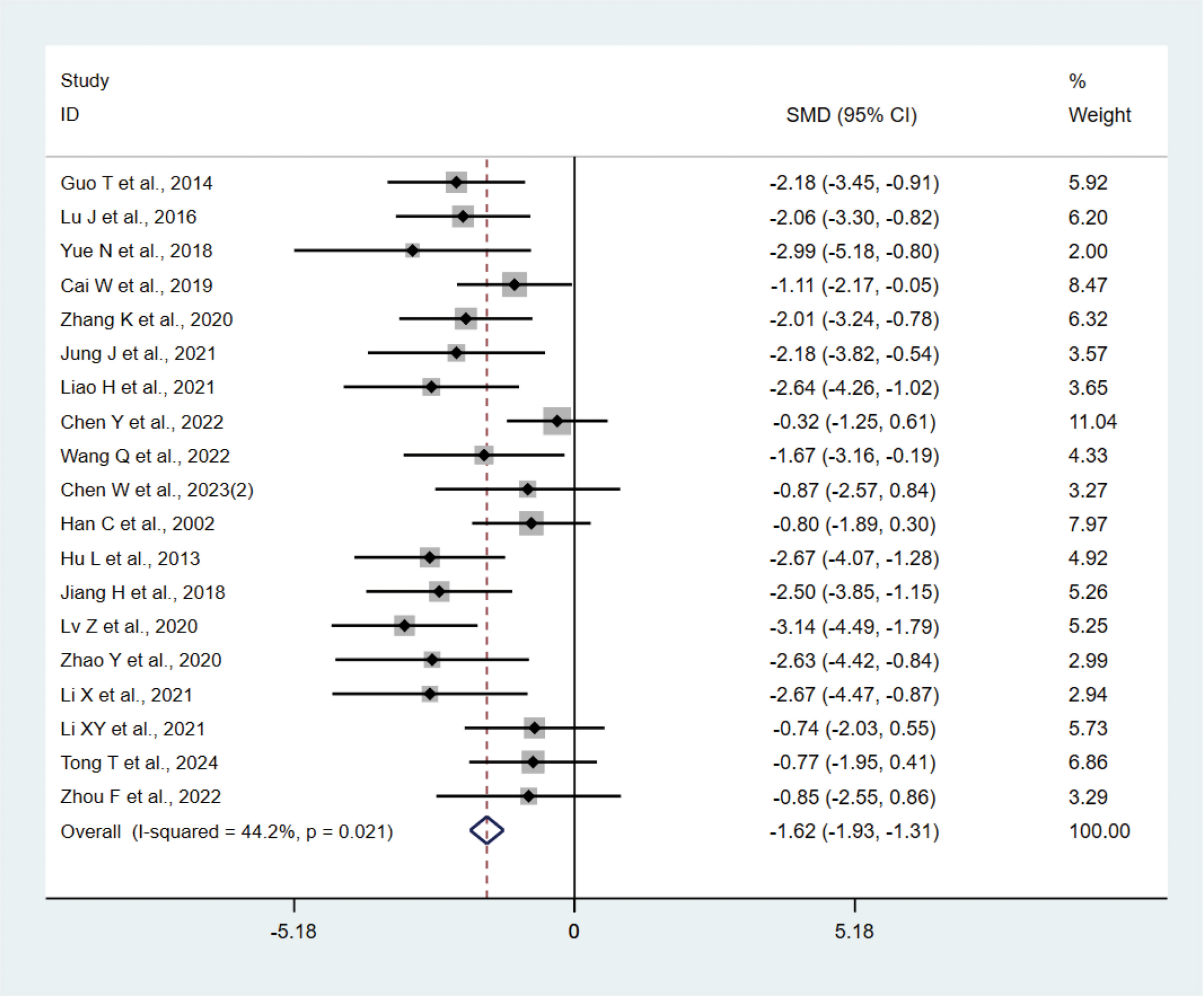
Meta-analysis of IL-1β.

A total of 19 studies (17, 20–22, 24, 25, 31, 36, 39, 43) with 242 animals involved IL-1β. Heterogeneity test showed that I²=44.2%, suggesting less heterogeneity, which was analyzed using a fixed-effect model. Meta-analysis showed that IL-1β was significantly lower in the test group than in the control group (SMD: −1.62, 95% CI: −1.93, −1.31), indicating that depression animals treated with acupuncture had significantly lower levels of IL-1β (P<0.05) (**Figure 4**). Sensitivity analysis showed stable results of meta-analysis (**Supplementary Figure 2**).

#### 3.4.2 IL-6

**Figure 5:**
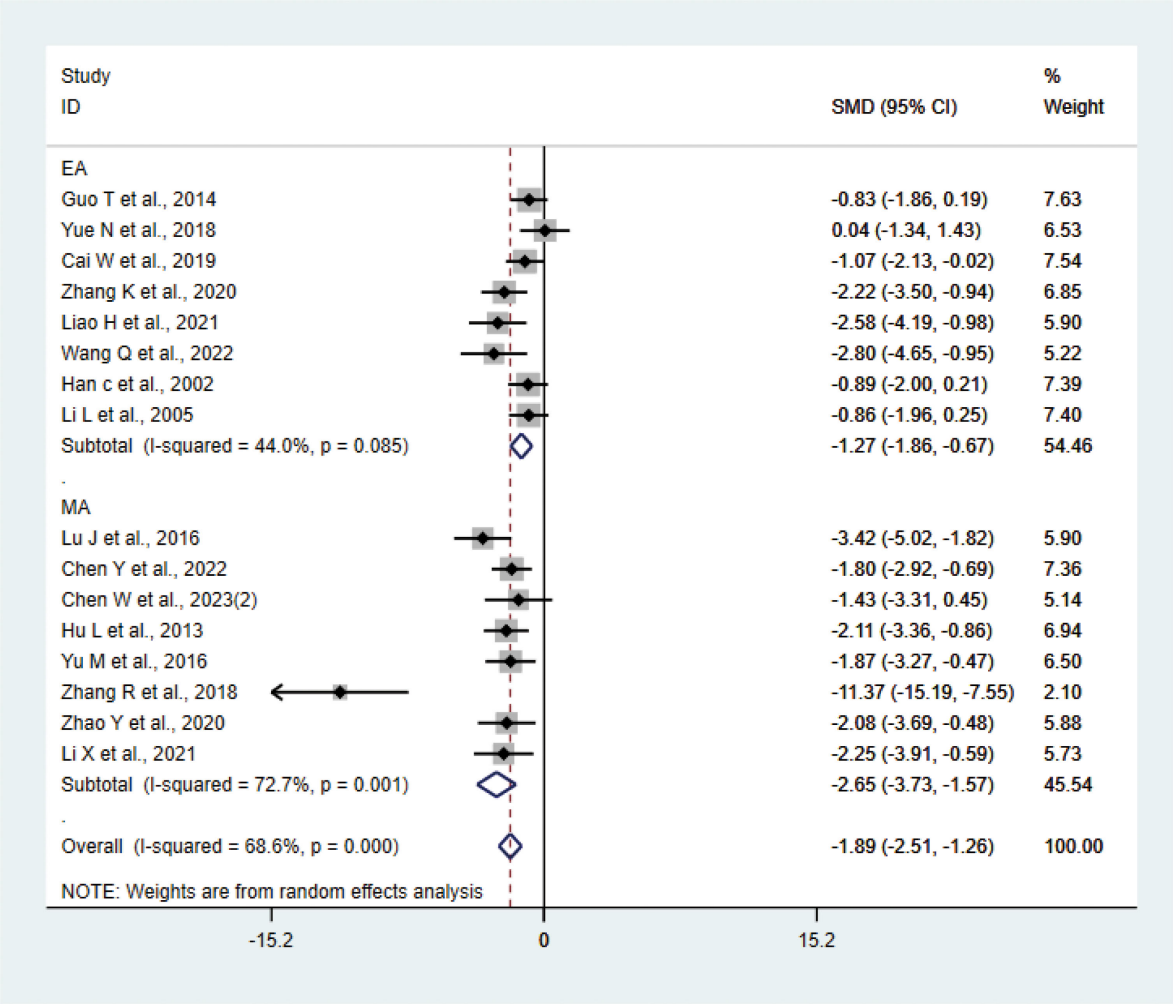
Meta-analysis of IL-6.

A total of 16 studies (17, 20, 21, 23, 27–29, 31, 33, 34, 36–38, 40–42) with 214 animals involved IL-6. Heterogeneity test showed that I²=68.6% suggesting moderate heterogeneity, which was analyzed using a random-effects model. Meta-analysis showed that IL-6 was significantly lower in the test group compared with the control group (SMD: −1.89, 95% CI: −2.51, −1.26), indicating that depression animals treated with acupuncture had significantly lower levels of IL-6 (P<0.05) (**Figure 5**). Sensitivity analysis showed stable results of meta-analysis (**Supplementary Figure 3**). We performed subgroup meta-analysis of different acupuncture methods to investigate heterogeneity (MA EA). Subgroup analysis showed that IL-6 was lower in the EA group than the control group (SMD: −1.27, 95% CI: −1.86, −0.67, I²=44.0%), and IL-6 was lower in the MA than in the control group (SMD: −2.65, 95% CI: −3.73, −1.57, I²=72.7%), P<0.05. The heterogeneity was decreased compared with the previous one, suggesting that the acupuncture methods might be the source of heterogeneity.

#### 3.4.3 TNF-α

**Figure 6:**
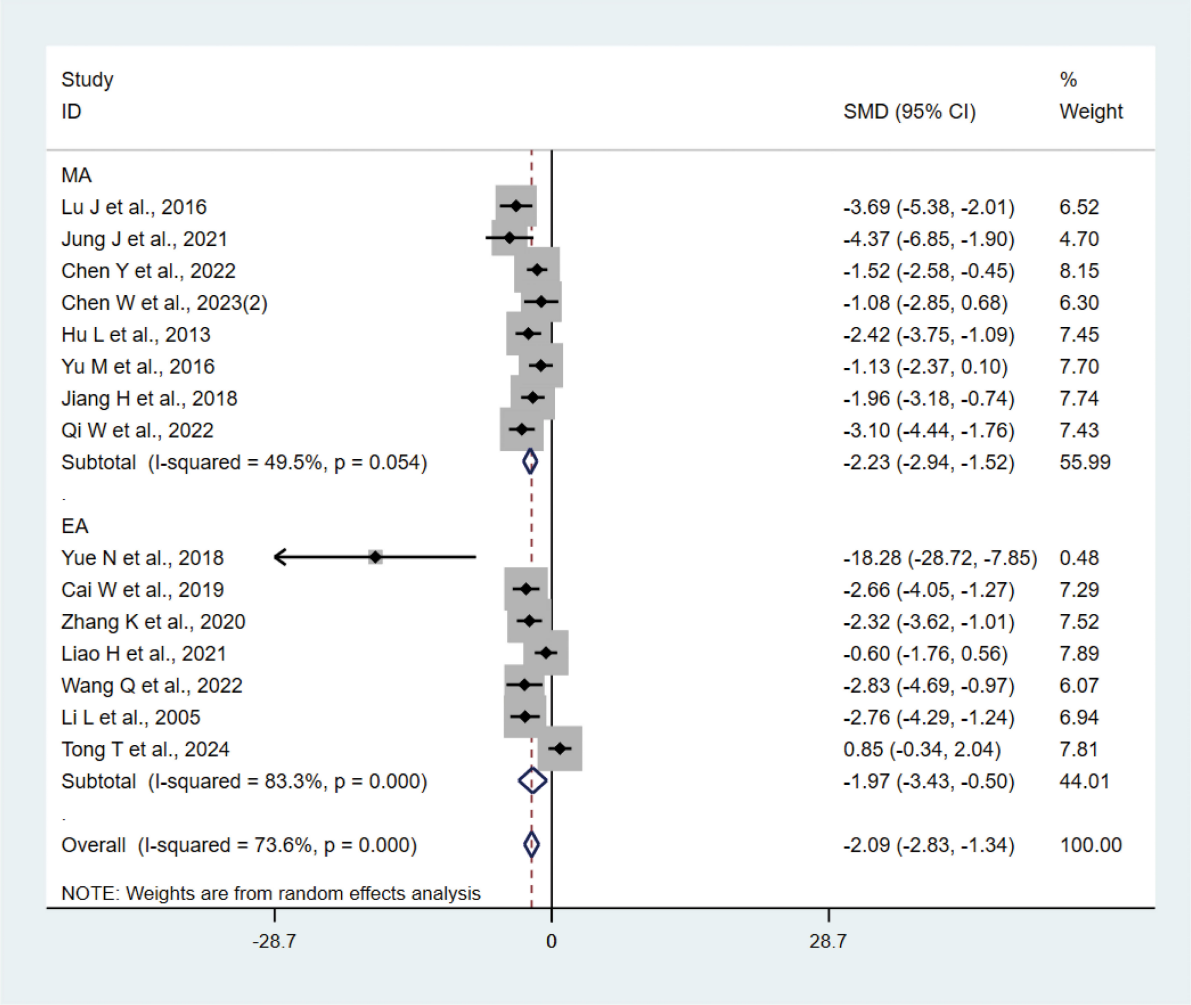
Meta-analysis of TNF-α.

A total of 15 studies (21–23, 27, 29, 31, 33, 35, 37, 38, 40–42) with 202 animals involved TNF-α. Heterogeneity test showed that I²=73.6%, suggesting moderate heterogeneity, which was analyzed using a random-effects model. Meta-analysis showed that TNF-α was lower in the test group compared with the control group (SMD: −2.09, 95% CI: −2.83, −1.34), indicating that depression animals treated with acupuncture had significantly lower levels of TNF-α (P<0.05) (**Figure 6**). Sensitivity analyses showed stable results from the meta-analysis (**Supplementary Figure 4**). To explore the source of heterogeneity, we performed subgroup meta-analysis of the different acupuncture methods (MA EA), and the results of the subgroup analysis showed that the MA had lower TNF-α than the control group (SMD: −2.23, 95% CI: −2.94, −1.52, I²=49.5%) and lower TNF-α in the EA than the control group (SMD: −1.97, 95% CI: −3.43, −0.50, I²=83.3%), P<0.05, with a decrease in heterogeneity compared with the previous one, suggesting that the acupuncture method may be the source of heterogeneity.

#### 3.4.4 IL-10

**Figure 7:**
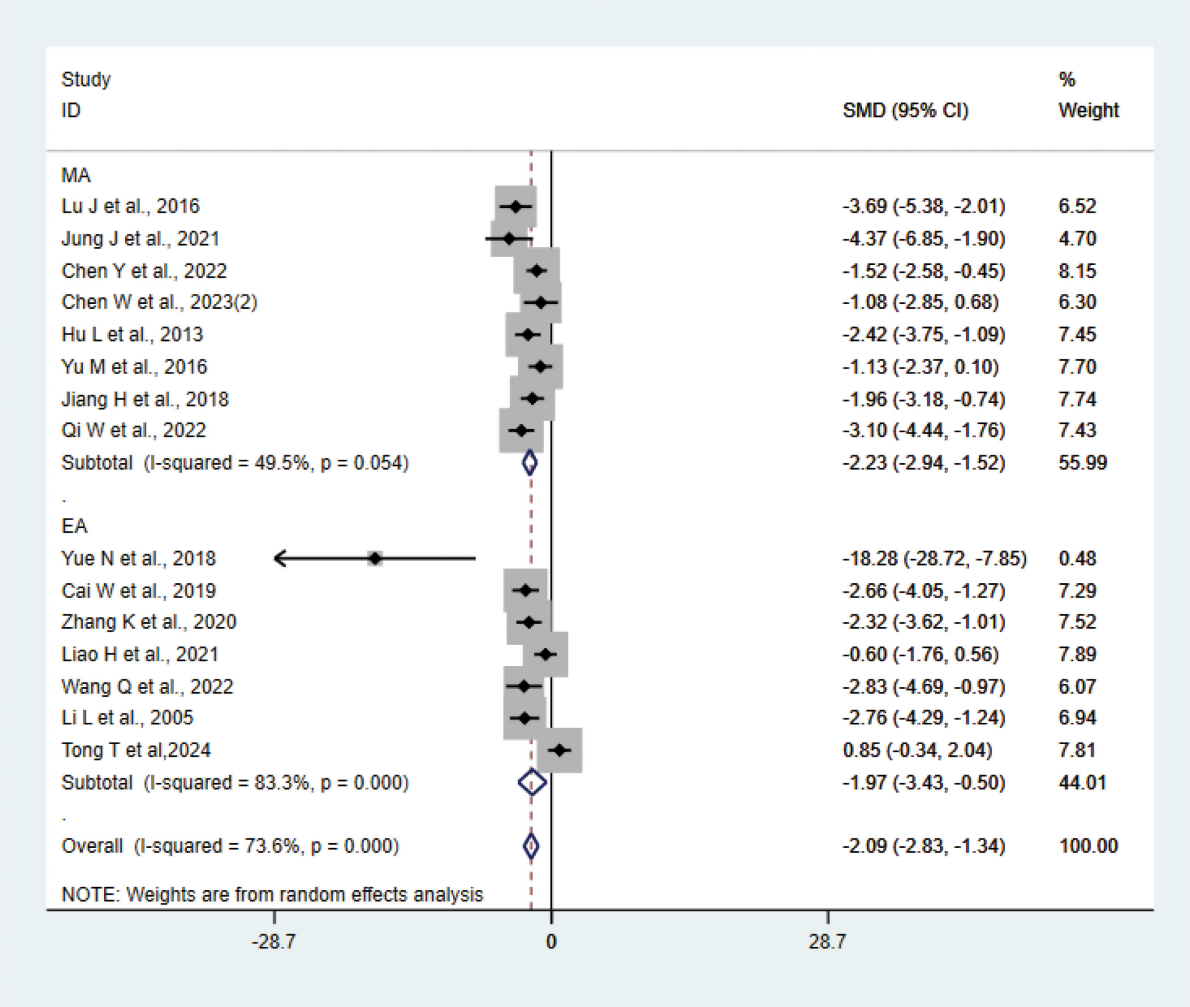
Meta-analysis of IL-10.

Five studies (17, 22, 25, 35, 37) with 68 animals involved IL-10. Heterogeneity test showed that I²=49.8%, suggesting less heterogeneity, which was analyzed using a fixed effect model. Meta-analysis showed that IL-10 was higher in the test group compared with the control group (SMD: 0.77, 95% CI:0.26, 1.28), indicating that depression animals treated with acupuncture had significantly higher levels of IL-10 (P < 0.05) (**Figure 7**). Sensitivity analysis showed stable results of meta-analysis (**Supplementary Figure 5**).

#### 3.4.5 IL-4

**Figure 8:**
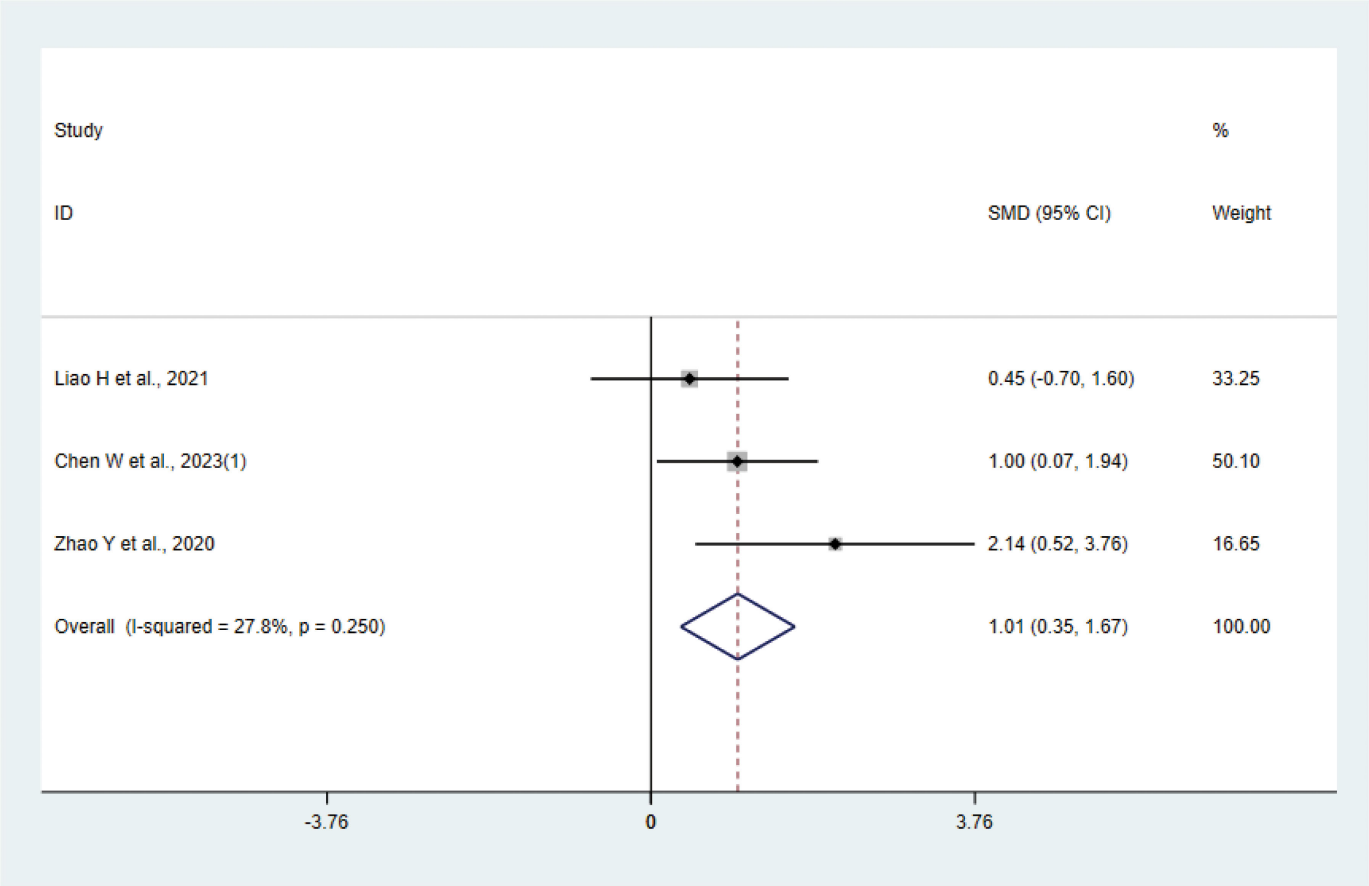
Meta-analysis of IL-4.

Three studies (17, 31, 37) with 42 animals involved IL-4. Heterogeneity test showed that I²=27.8% suggesting low heterogeneity, which was analyzed using a fixed effect model. Meta-analysis showed that IL-4 was higher in the test group than in the control group (SMD: 1.01, 95% CI: 0.35, 1.67), indicating that depression animals treated with acupuncture had significantly higher levels of IL-4 (P < 0.05) (**Figure 8**). Sensitivity analysis showed stable results of meta-analysis (**Supplementary Figure 6**).

#### 3.4.6 Body weight

**Figure 9:**
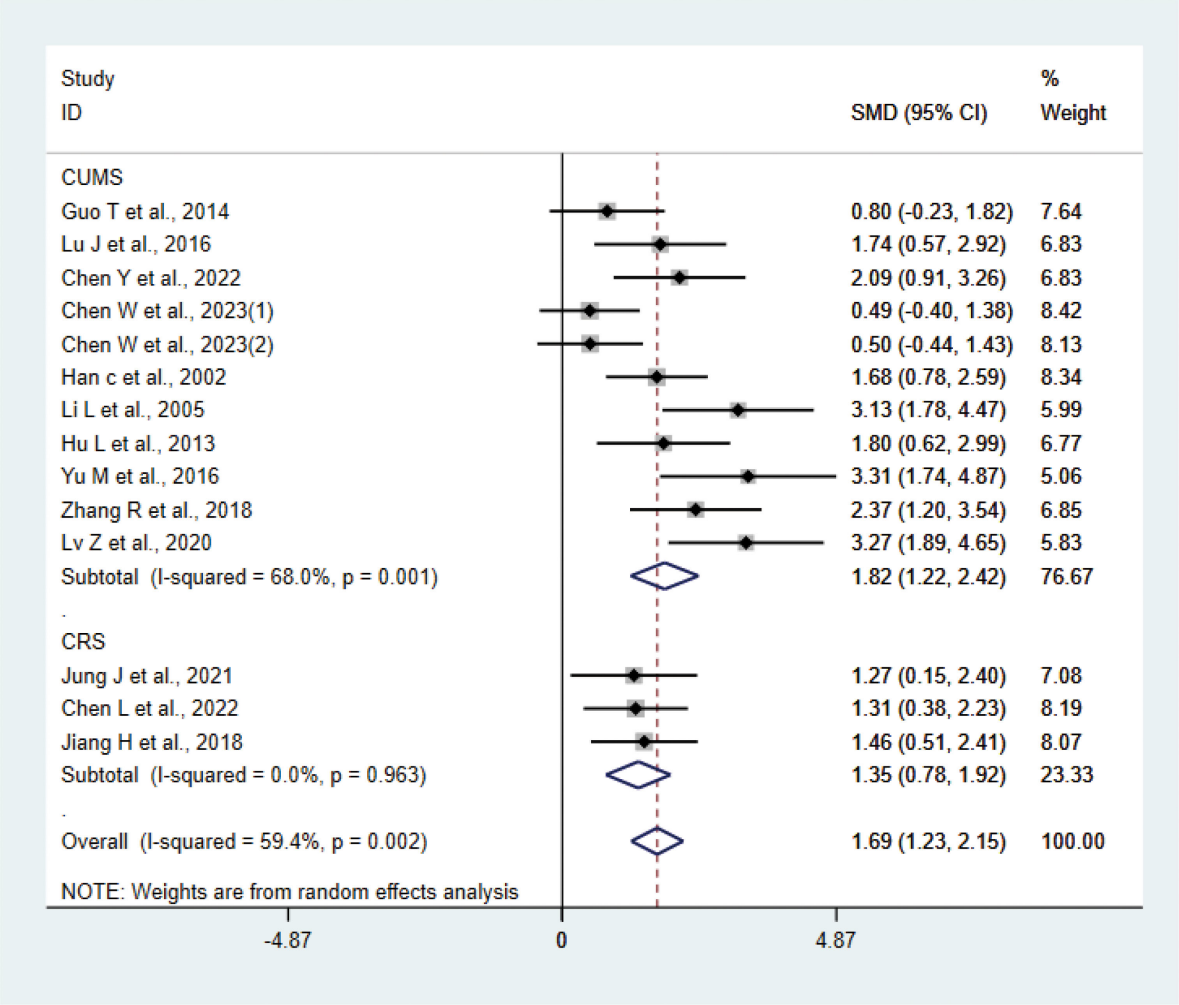
Meta-analysis of body weight.

A total of 14 studies (20–23, 25–28, 30, 31, 33, 34, 38, 39) with 265 animals involved body weight. The test for heterogeneity showed that I² = 59.4%, suggesting moderate heterogeneity, and was analyzed using a random effects model. Meta-analysis showed that the test group had heavier body weights than the control group (SMD: 1.69, 95% CI: 1.23, 2.15), suggesting that acupuncture significantly increased body weights of the depression animals (P < 0.05) (**Figure 9**). The results of the meta-analysis were stable according to sensitivity analysis (**Supplementary Figure 7**). To explore the source of heterogeneity, we performed subgroup meta-analysis of different modeling methods (CUMS CRS), and according to subgroup analysis, the test group in CRS was heavier than the control group in CRS (SMD: 1.35, 95% CI: 0.78, 1.92, I²=0.0%), and the test group was heavier than the control group in CUMS (SMD: 1.82, 95% CI: 1.22, 2.42, I²=68.0%), P<0.05. Heterogeneity was reduced compared with before, suggesting that the modeling methods may be the source of heterogeneity.

#### 3.4.7 OFT

**Figure 10:**
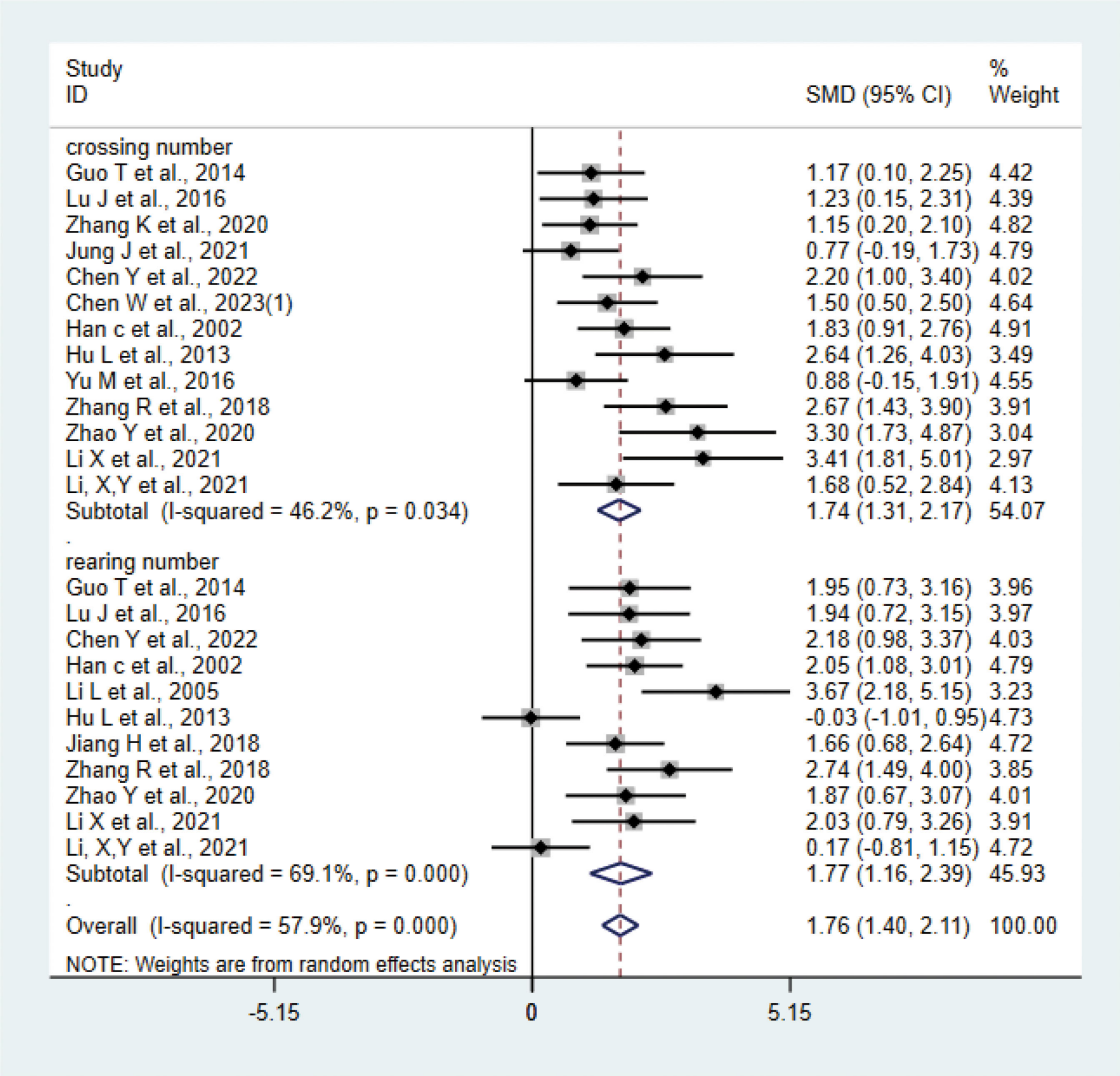
Meta-analysis of OFT.

A total of 15 studies (17, 20–24, 27, 28, 31, 33–36, 38, 42) with 276 animals were involved in OFT. The test for heterogeneity showed that I²=57.9% suggesting moderate heterogeneity, which was analyzed using a random-effects model. Based on the meta-analysis, it was found that the test group had a greater number of crossing numbers than the control group (SMD: 1.74, 95% CI: 1.31,2.17), indicating that acupuncture significantly increased the crossing number of depression animals (P<0.05) (**Figure 10**). The rearing numbers of the test group were more than those of the control group (SMD: 1.77, 95% CI: 1.16,2.39), indicating that acupuncture significantly increased the rearing number of depression animals (P < 0.05) (**Figure 10**). Sensitivity analysis showed stable results of meta-analysis (**Supplementary Figure 8**). Subgroup analysis of different acupuncture methods was conducted to explore the source of heterogeneity in rearing numbers (MA EA) (**Supplementary Figure 9**); as a result of subgroup meta-analysis, the EA rearing numbers were higher than those of the control group (SMD: 2.48, 95% CI: 1.77,3.18, I²=27.3%), and the MA rearing numbers were higher than those of the control group (SMD: 1.35, 95% CI: 0.63, 2.07, I²=66.5%), P<0.05. The heterogeneity was decreased compared with the previous one, suggesting that the acupuncture method may be the source of heterogeneity.

### 3.5 Publication bias

Inverted funnel plots and Egger’s test were used to detect publication bias for outcomes involving more than 10 studies. According to the Egger’s test, IL-1β, IL-6, TNF-α, body weight, and OFT showed a statistically significant publication bias (P<0.05), which indicated that these studies might have been biased by publication (**Supplementary Figure 10**). To assess the impact of publication bias (IL-1β, IL-6, TNF-α, body weight, and OFT) on the results, we applied the trimming and filling method. The results showed that the robustness of body weight and OFT was not significantly affected by publication bias (**Supplementary Table 3**). After trimming and filling, no studies were added to IL-1β, IL-6, and TNF-α. This may be because Egger’s test assumes publication bias as the sole cause of funnel plot asymmetry, overlooking other potential factors (differences in study design, sample selection bias). The trimming and filling method assumes that funnel chart asymmetry stems from publication bias, with missing studies symmetrically distributed around existing research. If actual asymmetry arises from causes other than publication bias, this method may fail to effectively fill gaps, resulting in no additional studies being incorporated.

### 3.6 Certainty assessment

Evidence quality was evaluated using GRADE (**Table 1**). Due to bias risks, IL-1 was assessed moderate; IL-6, TNF-α, body weight, and OFT were assessed as low due to the presence of risk of bias and heterogeneity. IL-10 and IL-4 were assessed as moderate because of small sample sizes.

**Table 1:** Certainty assessment.

Outcomes	Number of studies	Design	Risk of bias	Inconsistency	Indirectness	Imprecision	Other considerations	Number of animals	Certainty assessment	Effect	Quality
								Test group	Control group	SMD(95% CI)	
IL-1β	19	Randomized trials	Serious1	No serious inconsistency	No serious indirectness	No serious imprecision	No serious	121	121	−1.62 (−1.93,−1.31)	Moderate
IL-6	16	Randomized trials	Serious1	Serious2	No serious indirectness	No serious imprecision	No serious	107	107	−1.89 (−2.51,−1.26)	Low
TNF-α	15	Randomized trials	Serious1	Serious2	No serious indirectness	No serious imprecision	No serious	101	101	−2.09 (−2.83,−1.34)	Low
IL-10	5	Randomized trials	No serious inconsistency	No serious inconsistency	No serious indirectness	Serious3	No serious	34	34	0.77 (0.26, 1.28)	Moderate
IL-4	3	Randomized trials	No serious inconsistency	No serious inconsistency	No serious indirectness	Serious3	No serious	21	21	1.01 (0.35, 1.67)	Moderate
Body weight	14	Randomized trials	Serious1	Serious2	No serious indirectness	No serious imprecision	No serious	132	133	1.69 (1.23, 2.15)	Low
OFT	15	Randomized trials	Serious1	Serious2	No serious indirectness	No serious imprecision	No serious	138	138	1.76 (1.40, 2.15)	Low

1: Risk of bias in included studies.

2: Heterogeneity exists.

3: Smaller number of animals.

## 4 Discussion

The meta-analysis showed that acupuncture significantly reduced IL-1β, IL-6, and TNF-α levels; increased IL-4 and IL-10 levels; improved depressive-like behavior (OFT); and increased body weight in depressive animal models compared with the control group.

Statistical analysis revealed that most of the inflammatory markers measured in the included studies were derived from brain tissues such as the hippocampus, prefrontal cortex, and amygdala, with a small portion originating from serum. Some studies have shown that both serum inflammatory factors and central nervous system inflammatory factors are important contributors to depression (44), and serum inflammatory factors can disrupt the blood–brain barrier, entering the central nervous system and inducing or exacerbating inflammatory responses (45). Therefore, investigating changes in central nervous system inflammatory responses during depression is highly meaningful. Based on previous research, a meta-analysis validated the efficacy and mechanisms of acupuncture in depressive animal models, suggesting that acupuncture may treat depression by reducing central nervous system inflammation (46); however, the study did not comprehensively examine all relevant inflammatory markers nor did it delve deeply into their underlying mechanisms. Therefore, we explored a more comprehensive set of neuroinflammatory markers of depression animal models to analyze the effects of acupuncture on neuroinflammation and its potential mechanisms.

### 4.1 Possible mechanisms

Our findings indicate that acupuncture can reduce TNF-α and IL-1β levels while increasing IL-10 and IL-4 levels in depression animals. Recent studies have shown that excessive activation of microglia leading to the release of pro-inflammatory cytokines is considered an important mechanism in depression (47). Based on the different inflammatory mediators they secrete, microglia can be classified into two polarized phenotypes: M1-type (pro-inflammatory) and M2-type (anti-inflammatory). In the M1 phenotype, activated microglia secrete superoxide free radicals and pro-inflammatory factors (such as TNF-α, IL-1β), thereby damaging neurons; in the M2 phenotype, upon activation, MG secretes anti-inflammatory factors (such as IL-10 and IL-4) and transforming growth factor-β (TGF-β), which promote the repair of neuronal damage (48). Previous studies have reported that microglia in the brains of depression patients exhibit abnormal activation, with significantly elevated expression levels of pro-inflammatory cytokines IL-1β, IL-6, and TNF-α (49).

An increasing number of studies have confirmed that acupuncture can inhibit the excessive activation of microglia during the depression process, reduce pro-inflammatory cytokines, and increase anti-inflammatory factor levels, thereby alleviating depression-like behavior in animal models. Studies have found that acupuncture at the Baihui and Yintang acupoints can reduce Iba-1 (a microglial marker) levels of CUMS-induced depressed rats and decrease the expression of pro-inflammatory cytokines IL-1β and IL-6, thereby inhibiting microglial activation and alleviating depressive-like behavior (24). This is consistent with our findings on the effects of acupuncture on pro-inflammatory factors. Electroacupuncture can also regulate the activation of microglia in the subgranular zone of the dentate gyrus of the hippocampus, reducing the expression of NLRP3, caspase-1, and IL-1β genes (50). Another study (51) showed that acupuncture can upregulate the expression of Arg-1 protein (a marker of M2-type microglia) in perimenopausal depressive rats, promote the polarization of microglia toward the M2 type, and simultaneously increase the levels of IL-4 and IL-10, thereby improving depressive-like behavior. Acupuncture can also downregulate the levels of pro-inflammatory cytokines IL-1β and IL-18 in the hippocampus of CUMS-induced depressed rats and increase the level of the anti-inflammatory factor IL-10 (52). This is consistent with our findings on the effects of acupuncture on anti-inflammatory factors.

Our findings indicate that acupuncture reduces TNF-α and IL-1β levels in the brain tissue of depression animals; it may be due to the fact that acupuncture can regulate inflammatory cell signaling pathways. Specifically, acupuncture can alleviate central inflammatory damage by regulating the activation of inflammatory cell signaling pathways and reducing the release of inflammatory factors. Several studies support this view: Electroacupuncture can inhibit the NF-κB/NLRP3 pathway in the hippocampus of CUMS rats, reducing the expression of IL-6, IL-1β, IL-18, and TNF-α (40); acupuncture can reduce the expression of TLR4, MyD88, NF-κB, and TNF-α proteins in the hippocampus of rats subjected to chronic restraint stress, alleviating depressive behavior (53); and acupuncture can also reduce the expression of HMGB-1, Iba-1, and TNF-α in the hippocampus of rats with chronic restraint stress-induced depression, alleviating HMGB-1-mediated neuroinflammation (30). Additionally, Yue et al. (41) found that electroacupuncture intervention in CUMS rats led to reduced expression of P2X7R, IL-1β, TNF-α, and Iba-1 proteins in the hippocampus, suggesting that electroacupuncture may alleviate neuroinflammation through P2X7R-mediated signaling pathways and exert antidepressant effects.

**Figure 11:**
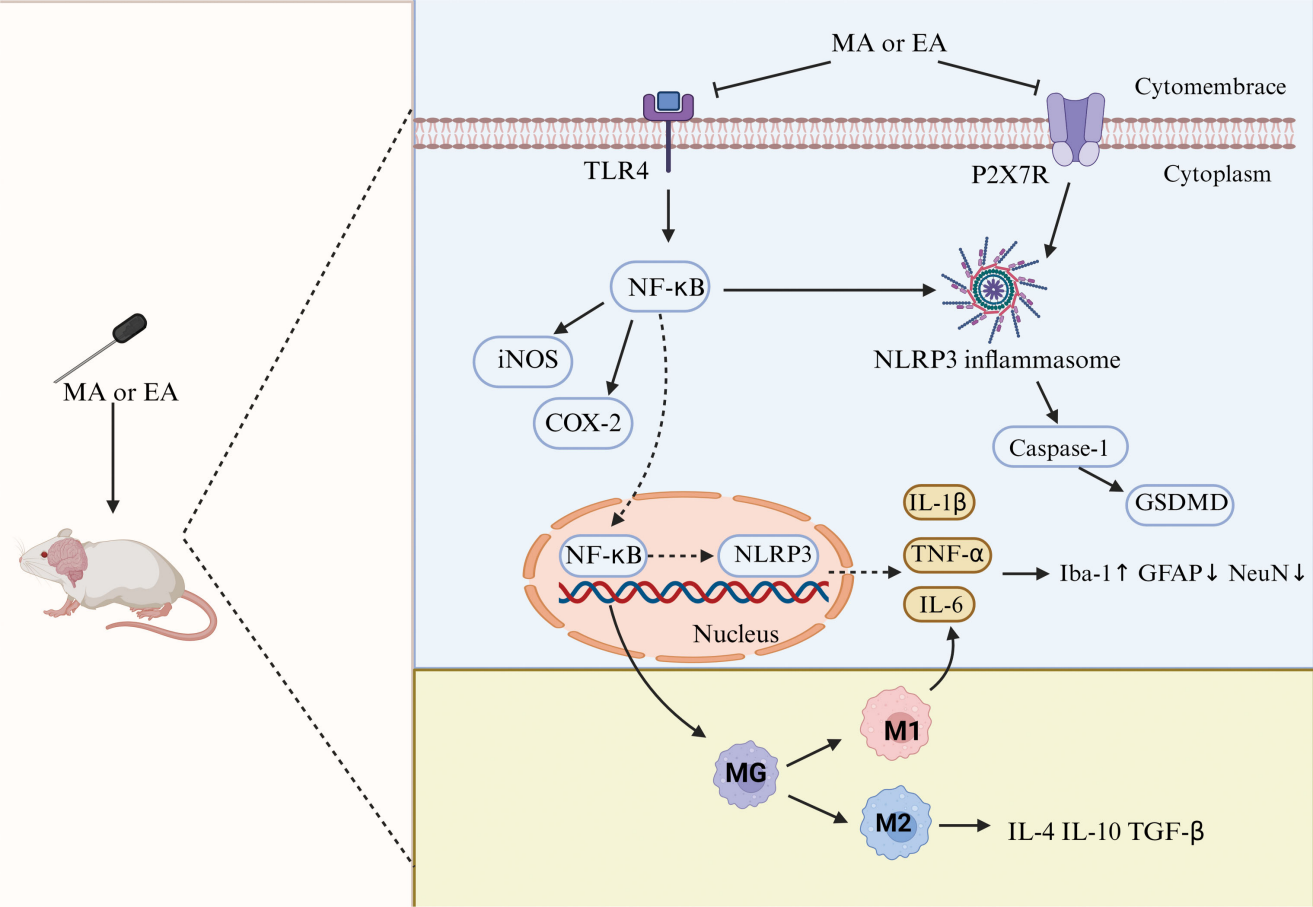
Neuroinflammatory mechanisms of antidepression in acupuncture. TLR4, toll-like receptor 4; NF-κB, nuclear factor kappa-B; iNOS, inducible nitric oxide synthase; COX-2, cyclooxygenase-2; NLRP3, NOD-, LRR-, and pyrin domain-containing protein 3; GSDMD, gasdermin D; GFAP, glial fibrillary acidic protein; NeuN, neuron-specific nuclear protein.

In summary, acupuncture influences the polarization of microglia, inhibits the polarization of MG toward the M1 type, reduces the release of pro-inflammatory factors, promotes the polarization of MG toward the M2 type, increases the release of anti-inflammatory factors, and dynamically rebalances inflammatory cytokines, thereby alleviating central inflammatory responses and protecting neurons. This may be a potential mechanism for acupuncture treatment of depression (**Figure 11**).

### 4.2 Clinical application

In this study, we also paid attention to the frequency of the use of different acupoints and EA parameters as well as their effects on inflammatory factors. In terms of the pattern of acupoints selection, we found that GV20 and GV29 were used with the highest frequency, which is consistent with the previous pattern of point selection in the treatment of depression with acupuncture (54). Study found that hippocampal NLRP3, caspase-1, and IL-1β protein expression was significantly reduced in depressed mice after EA GV20. The mechanism may be to downregulate the pro-inflammatory factors by inhibiting the activity of hippocampal NLRP3 inflammatory vesicles, thus reducing the neuroinflammatory responses (55). This suggests that GV20 is quite advantageous in central anti-inflammation; it may be the therapeutic mechanism of CV20 for depression. It is worth noting that we found SP6 and PC6 were also used with high frequency, which is different from the conventional acupoints for depression. It has been shown that acupuncture of SP6 can reduce muscle edema in mice of inflammatory muscle pain model, whereas this phenomenon was not found in IL-10 knockout mice, which is possible that acupuncture promotes IL-10 release and the transformation of macrophages from type M1 to type M2 (56, 57). The above studies have shown that SP6 is also effective in anti-inflammation, and its mechanism may be related to promoting the release of IL-10 to reduce inflammation in the central system. Therefore, in clinical practice, in addition to the conventional matching points of GV20 and GC29, the combination of SP6 may have a better therapeutic effect on the alleviation of depression. In terms of EA parameters, the sparse and dense waves of 2 Hz/100 Hz are used most frequently. Previous studies have found that stimulation of ST36 and ST25 with a frequency of 2 Hz/100 Hz with sparse waves can reduce the expression of local inflammatory factors in the intestinal tissues through the vagus nerve or the sympathetic nerve, respectively, and promote the restoration of gastrointestinal dynamics (58, 59). This suggests that the sparse-dense wave and 2 Hz/100 Hz may be more advantageous in reducing the inflammatory response. Therefore, in the treatment of depression, this frequency may be more effective at reducing neuroinflammatory responses. Furthermore, we have incorporated both EA and MA interventions, with research (60) suggesting that these may possess distinct molecular mechanisms in antidepressant treatment; for instance, EA may tend to alleviate depression by modulating macrophage polarization, whereas MA may tend to alleviate depression by regulating inflammatory pathways. Therefore, caution is warranted when extrapolating these findings to clinical practice. Future studies should compare them as separate interventions to elucidate their respective unique therapeutic mechanisms.

### 4.3 Limitations

(1) None of the included studies mentioned whether concealed grouping was performed, and none of them explicitly described the blinding method administered by the experimental researchers and animal keepers, which may have an impact on the evaluation of the behavioral outcomes (2); the sample sizes of the indexes about anti-inflammatory factors, such as IL-4 and IL-10, are relatively small, which may have a small-sample effect (3); due to the language limitation, we only included Chinese and English publications, and it was not possible to fully include all the relevant studies.

## 5 Conclusion

Acupuncture may alleviate depression by attenuating neuroinflammatory responses; its mechanism may be related to regulating the activation of microglia and inflammatory factors. For validation of this conclusion, more high-quality, large-sample, multi-mechanism studies are needed in the future.

## Data Availability

The datasets presented in this study can be found in online repositories. The names of the repository/repositories and accession number(s) can be found in the article/**Supplementary Material**.
