# Supplementary material for: Effect of acupuncture on neuroinflammatory responses in depression animals: a systematic review and meta-analysis
Source: Front Psychiatry. 2025 Oct 31;16:1624648. doi: 10.3389/fpsyt.2025.1624648 (PMC12617222; doi:10.3389/fpsyt.2025.1624648)
Supplement: Supplementary file 1 [file DataSheet1.pdf]

Supplementary Material

	Random sequence generation (selection bias)	Baseline characteristics (selection bias)	Allocation concealment (selection bias)	Random housing (performance bias)	Blinding of participants and personnel (performance bias)	Random outcome assessment (detection bias)	Blinding of outcome assessment (detection bias)	Incomplete outcome data (attrition bias)	Selective reporting (reporting bias)	Other bias
Cai,W et al.,2019	+	+	?	+	?	?	?	+	+	+
Chen,L et al.,2022	+	+	?	+	?	?	+	+	+	+
Chen,W et al.,2023(1)	?	+	?	+	?	?	?	+	+	+
Chen,W et al.,2023(2)	?	+	?	+	?	?	?	+	+	+
Chen,Y et al.,2022	?	+	?	+	?	?	?	+	+	+
Guo,T et al.,2014	?	+	?	+	?	?	?	+	+	+
Han,C et al.,2002	?	+	?	+	?	?	?	+	+	+
Hu,L et al.,2013	?	+	?	+	?	?	?	+	+	+
Jiang,H et al.,2018	+	+	?	+	?	+	+	+	+	+
Jung,J et al.,2021	+	+	?	+	?	?	?	+	+	+
Li,L et al.,2005	?	+	?	+	?	?	+	+	+	+
Li,X,Y et al.,2021	?	+	?	+	?	?	?	+	+	+
Li,X et al.,2021	?	+	?	+	?	?	+	+	+	+
Liao,H et al.,2021	?	+	?	+	?	?	?	+	+	+
Lu,J et al.,2015	?	+	?	+	?	?	?	+	+	+
Lv,Z et al.,2020	+	+	?	+	?	?	?	+	+	+
Qi,W et al.,2022	?	+	?	+	?	?	?	+	+	+
Tong,T et al.,2024	?	+	?	+	?	?	?	+	+	+
Wang,Q et al.,2022	+	+	?	+	?	?	?	+	+	+
Yu,M et al.,2016	+	+	?	+	?	?	+	+	+	+
Yue,N et al.,2018	+	+	?	+	?	?	?	+	+	+
Zhang,K et al.,2020	?	+	?	+	?	?	?	+	+	+
Zhang,R et al.,2018	?	+	?	+	?	?	?	+	+	+
Zhao,Y et al.,2020	?	+	?	+	?	?	+	+	+	+
Zhou,F et al.,2022	?	+	?	+	?	?	?	+	+	+

Supplementary Figure 1. Risk of bias.

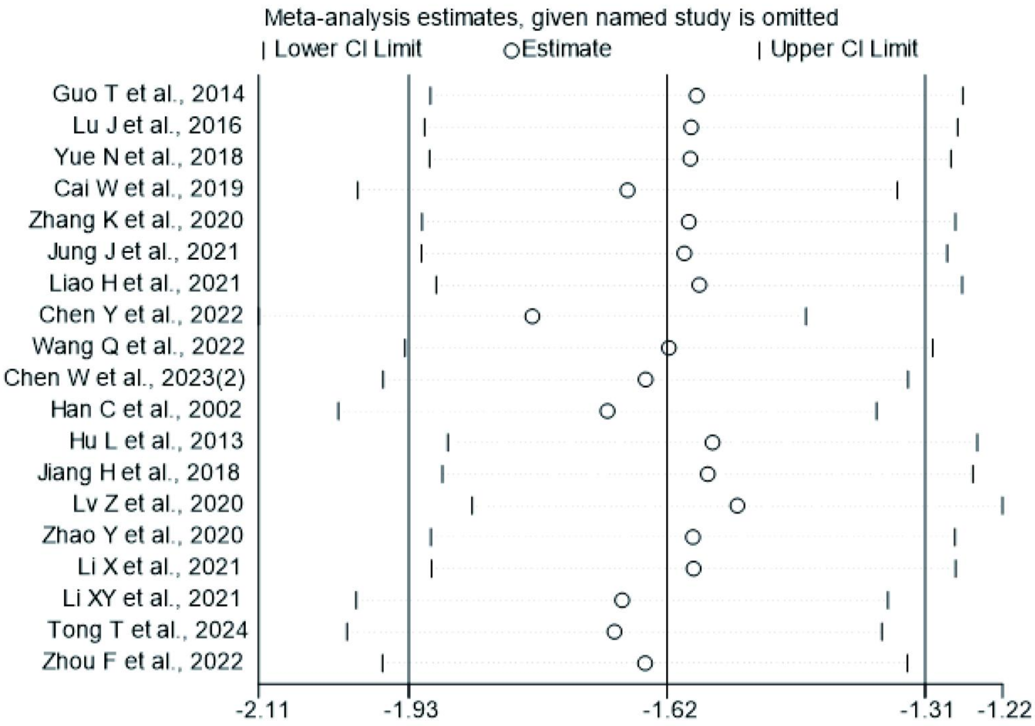

Supplementary Figure 2. Sensitivity analysis of IL-1β.

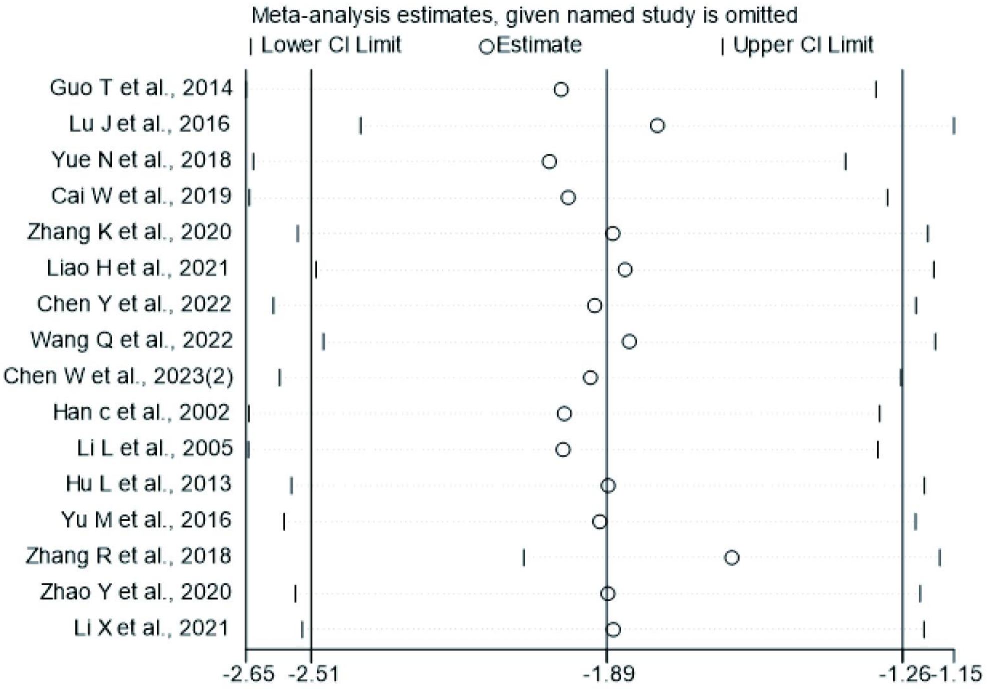

Supplementary Figure 3. Sensitivity analysis of IL-6.

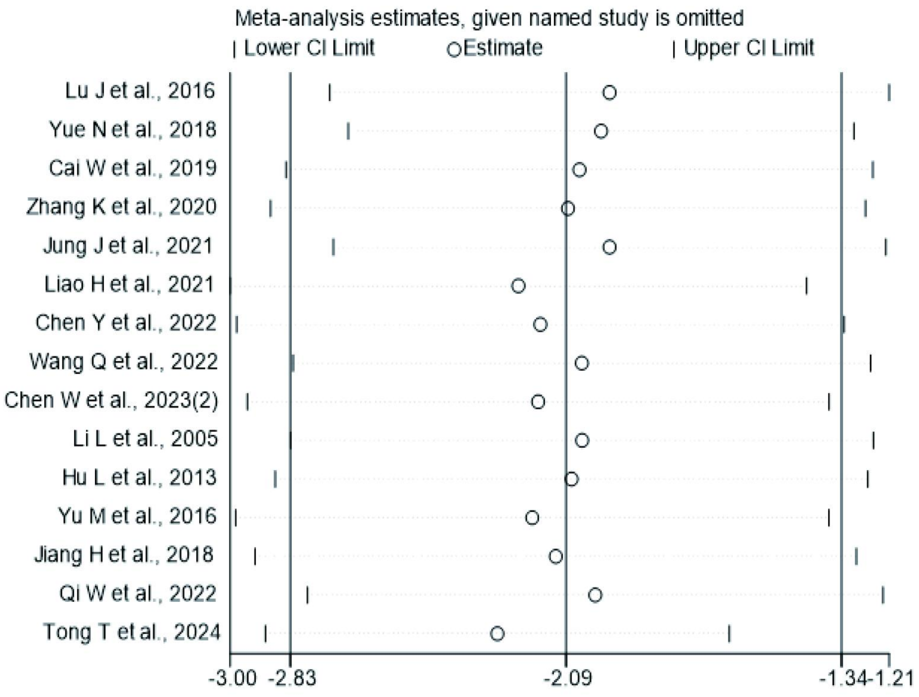

Supplementary Figure 4. Sensitivity analysis of TNF-α.

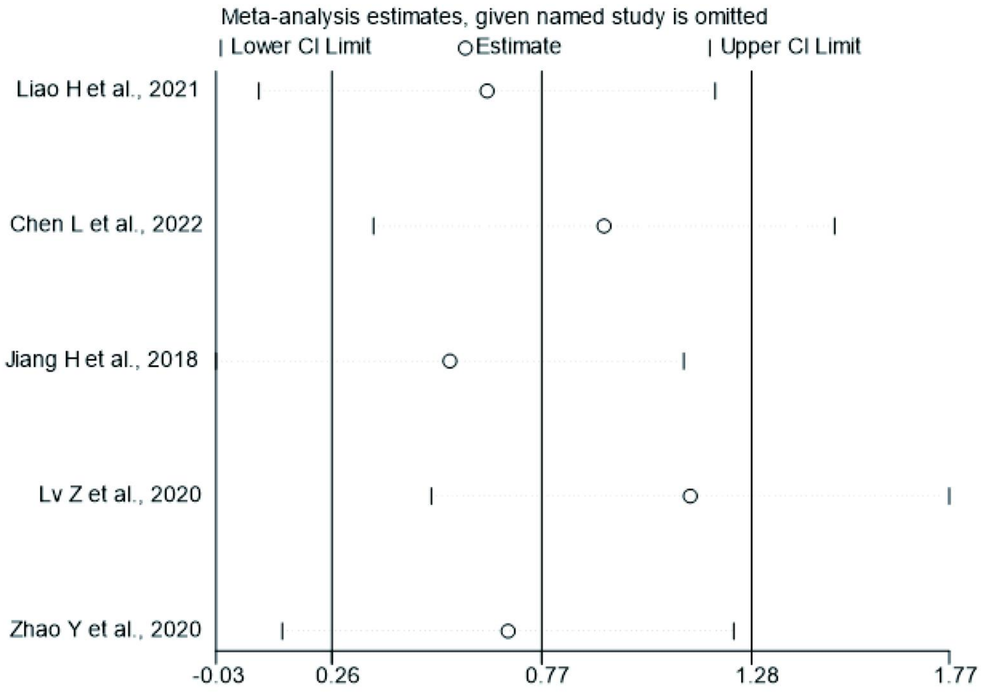

Supplementary Figure 5. Sensitivity analysis of IL-10.

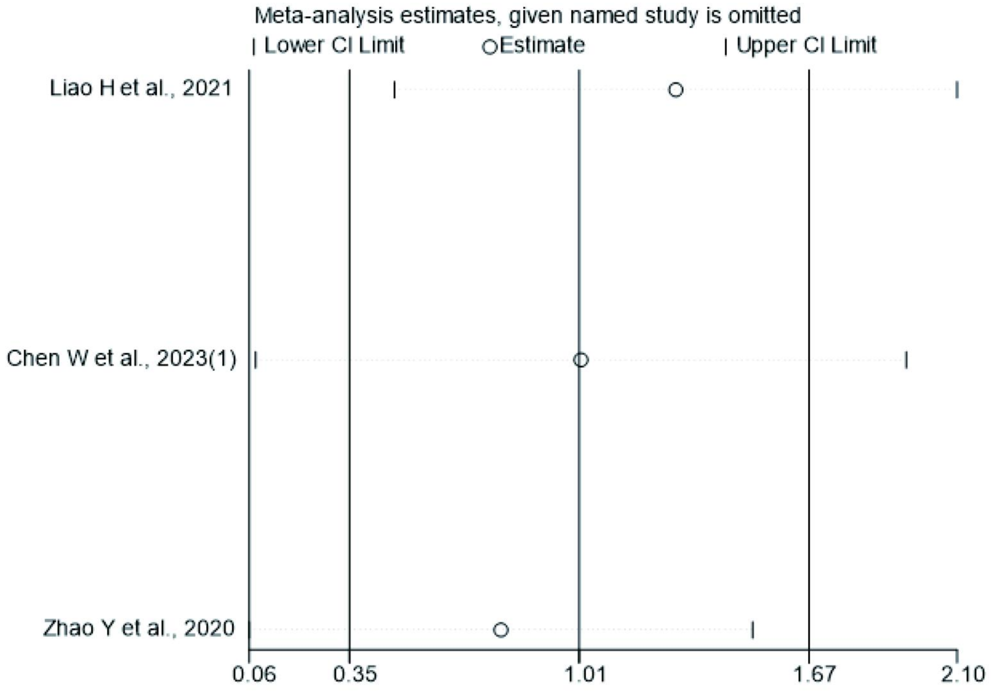

Supplementary Figure 6. Sensitivity analysis of IL-4.

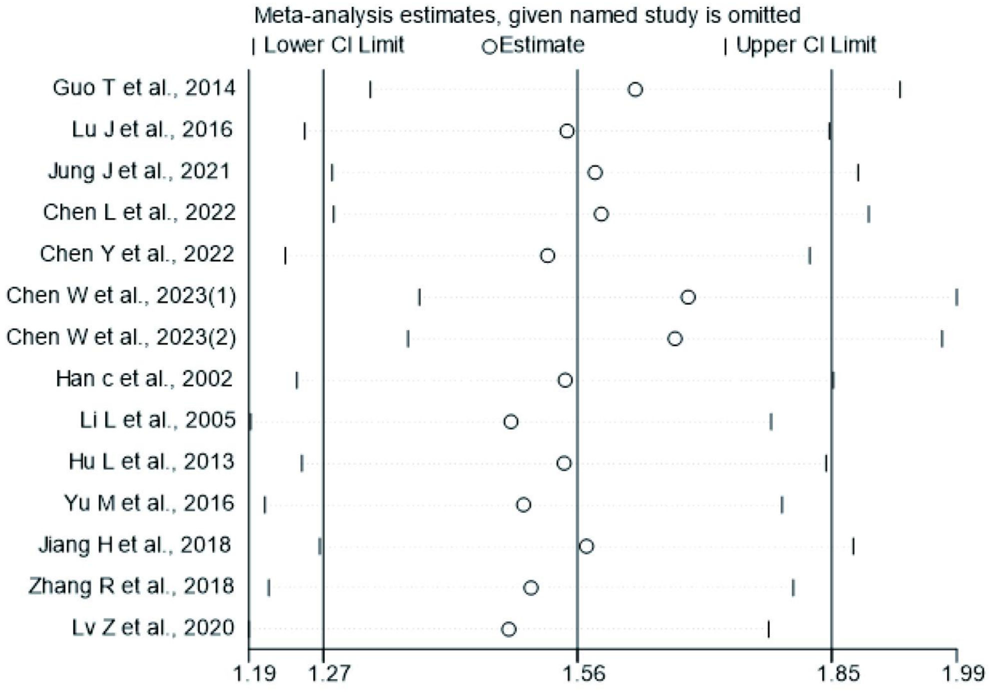

Supplementary Figure 7. Sensitivity analysis of body weight.

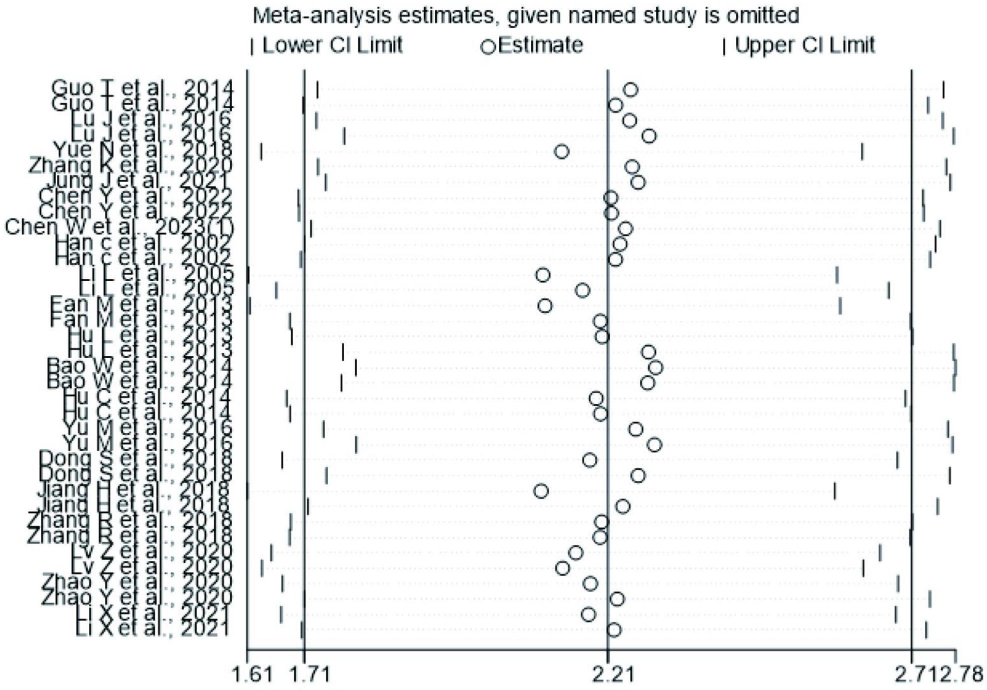

Supplementary Figure 8. Sensitivity analysis of OFT.

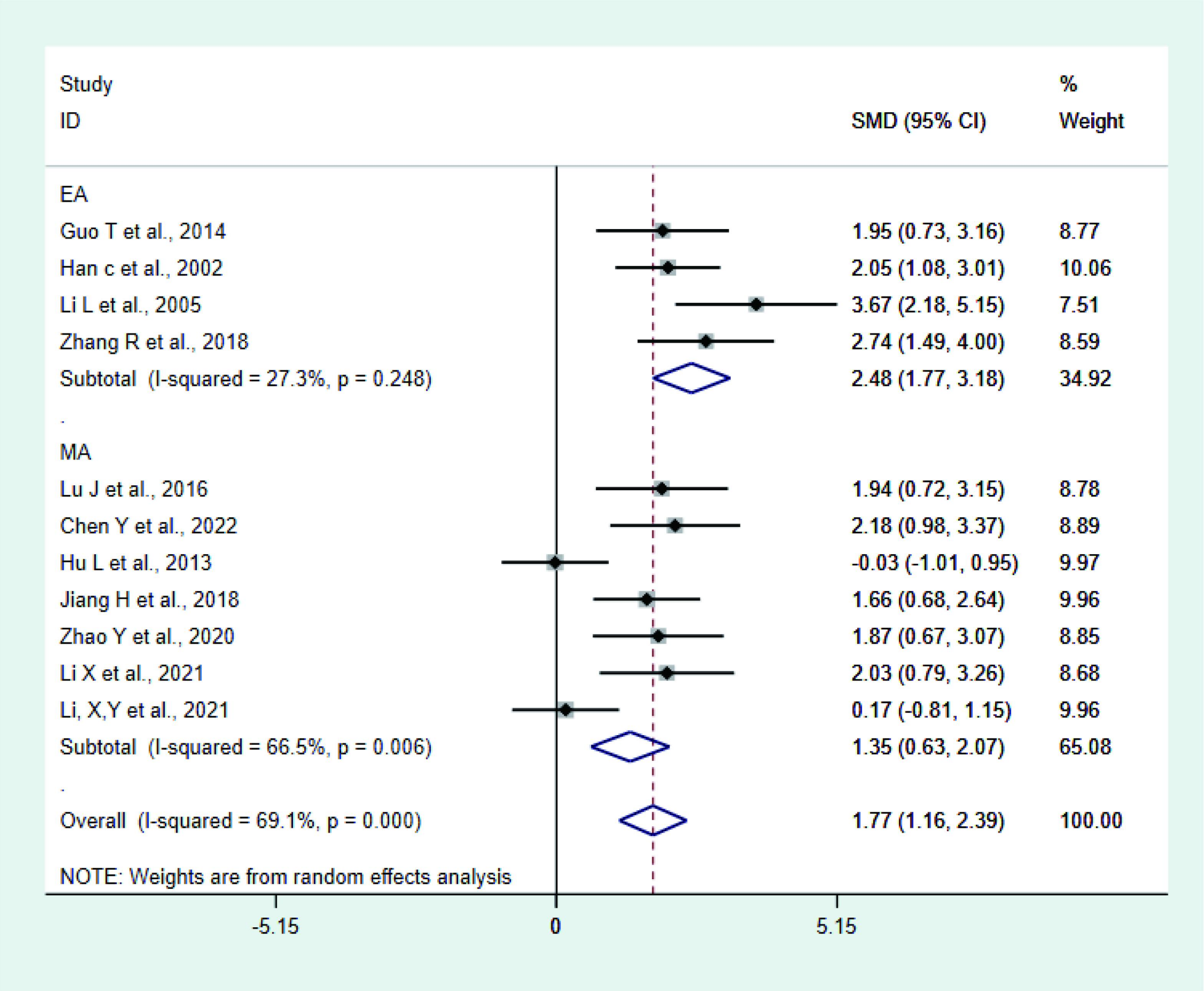

Supplementary Figure 9. Subgroup meta-analysis of OFT.

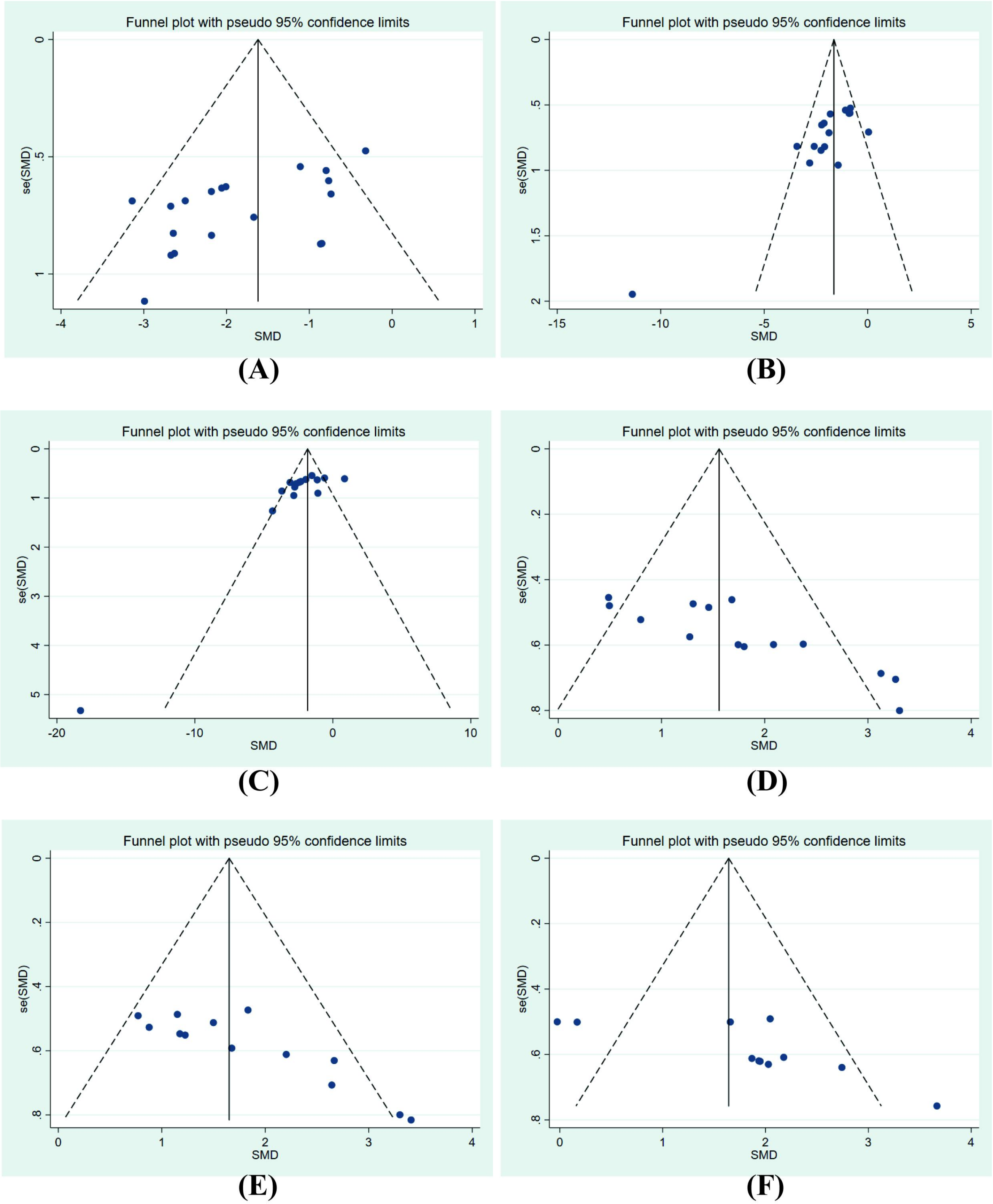

Supplementary Figure 10. Publication bias.
