# Supplementary material for: Effect of acupuncture on neuroinflammatory responses in depression animals: a systematic review and meta-analysis
Source: Front Psychiatry. 2025 Oct 31;16:1624648. doi: 10.3389/fpsyt.2025.1624648 (PMC12617222; doi:10.3389/fpsyt.2025.1624648)
Supplement: Supplementary file 2 [file Table1.docx]

Supplementary table 1: The search strategy.

**The search strategy (Pubmed)**

| Search number | Query | Results |
| --- | --- | --- |
| 1 | "acupuncture therapy"[MeSH Terms]) OR "electroacupuncture"[MeSH Terms] | 32181 |
| 2 | "abdominal needle"[Title/Abstract] OR "acupoint catgut embedding"[Title/Abstract] OR "acupoint catgut embedding therapy"[Title/Abstract] OR "acupoint injection"[Title/Abstract] OR "acupotom*"[Title/Abstract] OR "acupuncture"[Title/Abstract] OR "Acupuncture Therapy"[Title/Abstract] OR "acupuncture treatmen*"[Title/Abstract] OR "auricular acupunctur*"[Title/Abstract] OR "auriculo acupuncture"[Title/Abstract] OR "auriculoacupuncture"[Title/Abstract] OR "auriculotherapy"[Title/Abstract] OR "catgut embedding"[Title/Abstract] OR "catgut implantation"[Title/Abstract] OR "ear acupunctur*"[Title/Abstract] OR "electric acupuncture"[Title/Abstract] OR "electrical acupoint stimulation"[Title/Abstract] OR "electrical acupuncture"[Title/Abstract] OR "electro acupuncture"[Title/Abstract] OR "Electroacupuncture"[Title/Abstract] OR "electronic acupuncture"[Title/Abstract] OR "Pharmacoacupuncture Therapy"[Title/Abstract] OR "Pharmacoacupuncture Treatment"[Title/Abstract] | 36467 |
| 3 | "depressive disorder"[MeSH Terms] OR "depression"[MeSH Terms] OR "depressive disorder"[MeSH Terms] OR "depressive disorder, major"[MeSH Terms] | 284294 |
| 4 | "central depression"[Title/Abstract] OR "clinical depression"[Title/Abstract] OR "Depression"[Title/Abstract] OR "depression emotional"[Title/Abstract] OR "depression endogenous"[Title/Abstract] OR "depression involutional"[Title/Abstract] OR "depression major"[Title/Abstract] OR "depression unipolar"[Title/Abstract] OR "depressions endogenous"[Title/Abstract] OR "depressions unipolar"[Title/Abstract] OR "depressive disease"[Title/Abstract] OR "Depressive Disorder"[Title/Abstract] OR "depressive disorder major"[Title/Abstract] OR "Depressive Disorders"[Title/Abstract] OR "depressive disorders major"[Title/Abstract] OR "depressive episode"[Title/Abstract] OR "depressive illness"[Title/Abstract] OR "Depressive Neuroses"[Title/Abstract] OR "Depressive Neurosis"[Title/Abstract] OR "depressive personality disorder"[Title/Abstract] OR "depressive state"[Title/Abstract] OR "Depressive Symptom"[Title/Abstract] OR "Depressive Symptoms"[Title/Abstract] OR "Depressive Syndrome"[Title/Abstract] OR "Depressive Syndromes"[Title/Abstract] OR "depressivity"[Title/Abstract] OR "disorder depressive"[Title/Abstract] OR "disorders depressive"[Title/Abstract] OR "Emotional Depression"[Title/Abstract] OR "Endogenous Depression"[Title/Abstract] OR "Endogenous Depressions"[Title/Abstract] OR "Involutional Depression"[Title/Abstract] OR "Involutional Melancholia"[Title/Abstract] OR "Involutional Psychoses"[Title/Abstract] OR "Involutional Psychosis"[Title/Abstract] OR "major depression"[Title/Abstract] OR "Major Depressive Disorder"[Title/Abstract] OR "Major Depressive Disorders"[Title/Abstract] OR "major depressive episode"[Title/Abstract] OR "Melancholia"[Title/Abstract] OR "Melancholias"[Title/Abstract] OR "mental depression"[Title/Abstract] OR "neuroses depressive"[Title/Abstract] OR "neurosis depressive"[Title/Abstract] OR "Neurotic Depression"[Title/Abstract] OR "Neurotic Depressions"[Title/Abstract] OR "parental depression"[Title/Abstract] OR "psychoses involutional"[Title/Abstract] OR "psychosis involutional"[Title/Abstract] OR "symptom depressive"[Title/Abstract] OR "syndrome depressive"[Title/Abstract] OR "syndromes depressive"[Title/Abstract] OR "Unipolar Depression"[Title/Abstract] OR "Unipolar Depressions"[Title/Abstract] OR "unipolar disorder"[Title/Abstract] | 541707 |
| 5 | "animal experimentation"[MeSH Terms] OR "rats"[MeSH Terms] OR "mice"[MeSH Terms] OR "rabbits"[MeSH Terms] | 3800819 |
| 6 | "Animal Experiment"[Title/Abstract] OR "Animal Experimental Use"[Title/Abstract] OR "Animal Experimentation"[Title/Abstract] OR "Animal Experiments"[Title/Abstract] OR "animal physical conditioning"[Title/Abstract] OR "Animal Research"[Title/Abstract] OR "animal studies"[Title/Abstract] OR "animal study"[Title/Abstract] OR "animal trial"[Title/Abstract] OR "Belgian Hare"[Title/Abstract] OR "Chinchilla Rabbit"[Title/Abstract] OR "chronic unpredictable mild stress"[Title/Abstract] OR "CUMS"[Title/Abstract] OR "chronic restraint stress"[Title/Abstract] OR "CRS"[Title/Abstract] OR "Chinchilla Rabbits"[Title/Abstract] OR "cuniculus oryctolagus"[Title/Abstract] OR "Domestic Rabbit"[Title/Abstract] OR "Domestic Rabbits"[Title/Abstract] OR "domesticus mus musculus"[Title/Abstract] OR "experiment animal"[Title/Abstract] OR "experimentation animal"[Title/Abstract] OR "experiments animal"[Title/Abstract] OR "House Mouse"[Title/Abstract] OR "House Mice"[Title/Abstract] OR "Laboratory Mouse"[Title/Abstract] OR "Laboratory Rat"[Title/Abstract] OR "Laboratory Mice"[Title/Abstract] OR "Laboratory Rats"[Title/Abstract] OR "leporid"[Title/Abstract] OR "Leporidae"[Title/Abstract] OR "leporids"[Title/Abstract] OR "Mice"[Title/Abstract] OR "mice house"[Title/Abstract] OR "mice laboratory"[Title/Abstract] OR "mice swiss"[Title/Abstract] OR "Mouse"[Title/Abstract] OR "mouse house"[Title/Abstract] OR "mouse laboratory"[Title/Abstract] OR "mouse swiss"[Title/Abstract] OR "Mus"[Title/Abstract] OR "mus genus"[Title/Abstract] OR "Mus domesticus"[Title/Abstract] OR "Mus musculus"[Title/Abstract] OR "Mus musculus domesticus"[Title/Abstract] OR "New Zealand Rabbit"[Title/Abstract] OR "New Zealand White Rabbit"[Title/Abstract] OR "New Zealand White Rabbits"[Title/Abstract] OR "New Zealand Rabbits"[Title/Abstract] OR "newborn mice"[Title/Abstract] OR "norvegicus rattus"[Title/Abstract] OR "Norway Rat"[Title/Abstract] OR "Norway Rats"[Title/Abstract] OR "NZW Rabbit"[Title/Abstract] OR "NZW Rabbits"[Title/Abstract] OR "Oryctolagus cuniculus"[Title/Abstract] OR "Rabbit"[Title/Abstract] OR "rabbit chinchilla"[Title/Abstract] OR "rabbit domestic"[Title/Abstract] OR "rabbit new zealand"[Title/Abstract] OR "rabbit nzw"[Title/Abstract] OR "Rabbits"[Title/Abstract] OR "rabbits and hares"[Title/Abstract] OR "rabbits chinchilla"[Title/Abstract] OR "rabbits domestic"[Title/Abstract] OR "rabbits new zealand"[Title/Abstract] OR "rabbits nzw"[Title/Abstract] OR "Rat"[Title/Abstract] OR "rat laboratory"[Title/Abstract] OR "rat norway"[Title/Abstract] OR "Rats"[Title/Abstract] OR "rats laboratory"[Title/Abstract] OR "rats norway"[Title/Abstract] OR "Rattus"[Title/Abstract] OR "Rattus norvegicus"[Title/Abstract] OR "research animal"[Title/Abstract] OR "Swiss Mouse"[Title/Abstract] OR "Swiss Mice"[Title/Abstract] | 3336298 |
| 7 | (#1 OR #2) AND (#3 OR #4) AND (#5 OR #6) | 384 |

**The search strategy (Embase)**

| Search number | Query | Results |
| --- | --- | --- |
| 1 | 'acupuncture'/exp OR 'acupuncture' OR 'electroacupuncture'/exp OR 'electroacupuncture' | 82250 |
| 2 | 'depression'/exp OR 'major depression'/exp | 780028 |
| 3 | 'animal experiment'/exp OR 'rat'/exp OR 'mouse'/exp OR 'leporidae'/exp | 5630297 |
| 4 | 'abdominal needle':ab,ti,kw OR 'acet acupoint catgut embedding therapy':ab,ti,kw OR 'acupoint catgut embedding':ab,ti,kw OR 'acupoint catgut embedding therapy':ab,ti,kw OR 'acupoint injection':ab,ti,kw OR 'acupotom*':ab,ti,kw OR 'acupuncture':ab,ti,kw OR 'acupuncture therapy':ab,ti,kw OR 'acupuncture treatmen*':ab,ti,kw OR 'auricular acupunctur*':ab,ti,kw OR 'auriculo acupuncture':ab,ti,kw OR 'auriculoacupuncture':ab,ti,kw OR 'auriculotherapy':ab,ti,kw OR 'catgut embedding':ab,ti,kw OR 'catgut implantation':ab,ti,kw OR 'ear acupunctur*':ab,ti,kw OR 'earlobe acupuncture':ab,ti,kw OR 'electric acupuncture':ab,ti,kw OR 'electrical acupoint stimulation':ab,ti,kw OR 'electrical acupuncture':ab,ti,kw OR 'electro acupuncture':ab,ti,kw OR 'electroacupuncture':ab,ti,kw OR 'electrode acupuncture':ab,ti,kw OR 'electronic acupuncture':ab,ti,kw OR 'pharmacoacupuncture therapy':ab,ti,kw OR 'pharmacoacupuncture treatment':ab,ti,kw | 52677 |
| 5 | 'central depression':ab,ti,kw OR 'clinical depression':ab,ti,kw OR 'depression':ab,ti,kw OR 'depression, emotional':ab,ti,kw OR 'depression, endogenous':ab,ti,kw OR 'depression, involutional':ab,ti,kw OR 'depression, major':ab,ti,kw OR 'depression, unipolar':ab,ti,kw OR 'depressions, endogenous':ab,ti,kw OR 'depressions, neurotic':ab,ti,kw OR 'depressions, unipolar':ab,ti,kw OR 'depressive disease':ab,ti,kw OR 'depressive disorder':ab,ti,kw OR 'depressive disorder, major':ab,ti,kw OR 'depressive disorders':ab,ti,kw OR 'depressive disorders, major':ab,ti,kw OR 'depressive episode':ab,ti,kw OR 'depressive illness':ab,ti,kw OR 'depressive neuroses':ab,ti,kw OR 'depressive neurosis':ab,ti,kw OR 'depressive personality disorder':ab,ti,kw OR 'depressive state':ab,ti,kw OR 'depressive symptom':ab,ti,kw OR 'depressive symptoms':ab,ti,kw OR 'depressive syndrome':ab,ti,kw OR 'depressive syndromes':ab,ti,kw OR 'depressivity':ab,ti,kw OR 'disorder, depressive':ab,ti,kw OR 'disorders, depressive':ab,ti,kw OR 'emotional depression':ab,ti,kw OR 'endogenous depression':ab,ti,kw OR 'endogenous depressions':ab,ti,kw OR 'involutional depression':ab,ti,kw OR 'involutional melancholia':ab,ti,kw OR 'involutional paraphrenia':ab,ti,kw OR 'involutional paraphrenias':ab,ti,kw OR 'involutional psychoses':ab,ti,kw OR 'involutional psychosis':ab,ti,kw OR 'major depression':ab,ti,kw OR 'major depressive disorder':ab,ti,kw OR 'major depressive disorders':ab,ti,kw OR 'major depressive episode':ab,ti,kw OR 'melancholia':ab,ti,kw OR 'melancholia, involutional':ab,ti,kw OR 'melancholias':ab,ti,kw OR 'mental depression':ab,ti,kw OR 'neuroses, depressive':ab,ti,kw OR 'neurosis, depressive':ab,ti,kw OR 'neurotic depression':ab,ti,kw OR 'neurotic depressions':ab,ti,kw OR 'paraphrenia, involutional':ab,ti,kw OR 'paraphrenias, involutional':ab,ti,kw OR 'parental depression':ab,ti,kw OR 'psychoses, involutional':ab,ti,kw OR 'psychosis, involutional':ab,ti,kw OR 'symptom, depressive':ab,ti,kw OR 'syndrome, depressive':ab,ti,kw OR 'syndromes, depressive':ab,ti,kw OR 'unipolar depression':ab,ti,kw OR 'unipolar depressions':ab,ti,kw OR 'unipolar disorder':ab,ti,kw | 767525 |
| 6 | 'animal experiment':ab,ti,kw OR 'animal experimental use':ab,ti,kw OR 'animal experimental uses':ab,ti,kw OR 'animal experimentation':ab,ti,kw OR 'animal experiments':ab,ti,kw OR 'animal physical conditioning':ab,ti,kw OR 'animal research':ab,ti,kw OR 'animal studies':ab,ti,kw OR 'animal study':ab,ti,kw OR 'animal trial':ab,ti,kw OR 'belgian hare':ab,ti,kw OR 'chinchilla rabbit':ab,ti,kw OR 'chronic unpredictable mild stress':ab,ti,kw OR 'cums':ab,ti,kw OR 'chronic restraint stress':ab,ti,kw OR 'crs':ab,ti,kw OR 'chinchilla rabbits':ab,ti,kw OR 'cuniculus, oryctolagus':ab,ti,kw OR 'domestic rabbit':ab,ti,kw OR 'domestic rabbits':ab,ti,kw OR 'domesticus, mus musculus':ab,ti,kw OR 'experiment, animal':ab,ti,kw OR 'experimental use, animal':ab,ti,kw OR 'experimental uses, animal':ab,ti,kw OR 'experimentation, animal':ab,ti,kw OR 'experiments, animal':ab,ti,kw OR 'hare, belgian':ab,ti,kw OR 'house mouse':ab,ti,kw OR 'house mice':ab,ti,kw OR 'laboratory mouse':ab,ti,kw OR 'laboratory rat':ab,ti,kw OR 'laboratory mice':ab,ti,kw OR 'laboratory rats':ab,ti,kw OR 'leporid':ab,ti,kw OR 'leporidae':ab,ti,kw OR 'leporids':ab,ti,kw OR 'mice':ab,ti,kw OR 'mice, house':ab,ti,kw OR 'mice, laboratory':ab,ti,kw OR 'mice, swiss':ab,ti,kw OR 'mouse':ab,ti,kw OR 'mouse, house':ab,ti,kw OR 'mouse, laboratory':ab,ti,kw OR 'mouse, swiss':ab,ti,kw OR 'mus':ab,ti,kw OR 'mus (genus)':ab,ti,kw OR 'mus domesticus':ab,ti,kw OR 'mus musculus':ab,ti,kw OR 'mus musculus domesticus':ab,ti,kw OR 'new zealand rabbit':ab,ti,kw OR 'new zealand white rabbit':ab,ti,kw OR 'new zealand white rabbits':ab,ti,kw OR 'new zealand rabbits':ab,ti,kw OR 'newborn mice':ab,ti,kw OR 'norvegicus, rattus':ab,ti,kw OR 'norway rat':ab,ti,kw OR 'norway rats':ab,ti,kw OR 'nzw rabbit':ab,ti,kw OR 'nzw rabbits':ab,ti,kw OR 'oryctolagus cuniculus':ab,ti,kw OR 'physical conditioning, animal':ab,ti,kw OR 'rabbit':ab,ti,kw OR 'rabbit, chinchilla':ab,ti,kw OR 'rabbit, domestic':ab,ti,kw OR 'rabbit, new zealand':ab,ti,kw OR 'rabbit, nzw':ab,ti,kw OR 'rabbits':ab,ti,kw OR 'rabbits and hares':ab,ti,kw OR 'rabbits, chinchilla':ab,ti,kw OR 'rabbits, domestic':ab,ti,kw OR 'rabbits, new zealand':ab,ti,kw OR 'rabbits, nzw':ab,ti,kw OR 'rat':ab,ti,kw OR 'rat, laboratory':ab,ti,kw OR 'rat, norway':ab,ti,kw OR 'rats':ab,ti,kw OR 'rats, laboratory':ab,ti,kw OR 'rats, norway':ab,ti,kw OR 'rattus':ab,ti,kw OR 'rattus norvegicus':ab,ti,kw OR 'research, animal':ab,ti,kw OR 'swiss mouse':ab,ti,kw OR 'swiss mice':ab,ti,kw OR 'zealand rabbit, new':ab,ti,kw OR 'zealand rabbits, new':ab,ti,kw | 4209782 |
| 7 | (#1 OR #4) AND (#2 OR #5) AND (#3 OR #6) | 216 |

**The search strategy (Cochrane Library)**

| Search number | Query | Results |
| --- | --- | --- |
| 1 | MeSH descriptor: [Acupuncture Therapy] explode all trees | 13126 |
| 2 | MeSH descriptor: [Electroacupuncture] explode all trees | 4166 |
| 3 | ('abdominal needle' OR 'ACET acupoint catgut embedding therapy' OR 'acupoint catgut embedding' OR 'acupoint catgut embedding therapy' OR 'acupoint injection' OR 'Acupotom*' OR 'acupuncture' OR 'Acupuncture Therapy' OR 'Acupuncture Treatmen*' OR 'auricular acupunctur*' OR 'auriculo acupuncture' OR 'auriculoacupuncture' OR 'auriculotherapy' OR 'catgut embedding' OR 'catgut implantation' OR 'Ear Acupunctur*' OR 'earlobe acupuncture' OR 'electric acupuncture' OR 'electrical acupoint stimulation' OR 'electrical acupuncture' OR 'electro acupuncture' OR 'Electroacupuncture' OR 'electrode acupuncture' OR 'electronic acupuncture' OR 'Pharmacoacupuncture Therapy' OR 'Pharmacoacupuncture Treatment'):ab,ti,kw | 25371 |
| 4 | MeSH descriptor: [Depression] explode all trees | 116777 |
| 5 | MeSH descriptor: [Depressive Disorder] explode all trees | 27758 |
| 6 | ('central depression' OR 'clinical depression' OR 'Depression' OR 'Depression, Emotional' OR 'Depression, Endogenous' OR 'Depression, Involutional' OR 'depression, major' OR 'Depression, Unipolar' OR 'Depressions, Endogenous' OR 'Depressions, Neurotic' OR 'Depressions, Unipolar' OR 'depressive disease' OR 'Depressive Disorder' OR 'depressive disorder, major' OR 'Depressive Disorders' OR 'Depressive Disorders, Major' OR 'depressive episode' OR 'depressive illness' OR 'Depressive Neuroses' OR 'Depressive Neurosis' OR 'depressive personality disorder' OR 'depressive state' OR 'Depressive Symptom' OR 'Depressive Symptoms' OR 'Depressive Syndrome' OR 'Depressive Syndromes' OR 'depressivity' OR 'Disorder, Depressive' OR 'Disorders, Depressive' OR 'Emotional Depression' OR 'Endogenous Depression' OR 'Endogenous Depressions' OR 'Involutional Depression' OR 'Involutional Melancholia' OR 'Involutional Paraphrenia' OR 'Involutional Paraphrenias' OR 'Involutional Psychoses' OR 'Involutional Psychosis' OR 'major depression' OR 'Major Depressive Disorder' OR 'Major Depressive Disorders' OR 'major depressive episode' OR 'Melancholia' OR 'Melancholia, Involutional' OR 'Melancholias' OR 'mental depression' OR 'Neuroses, Depressive' OR 'Neurosis, Depressive' OR 'Neurotic Depression' OR 'Neurotic Depressions' OR 'Paraphrenia, Involutional' OR 'Paraphrenias, Involutional' OR 'parental depression' OR 'Psychoses, Involutional' OR 'Psychosis, Involutional' OR 'Symptom, Depressive' OR 'Syndrome, Depressive' OR 'Syndromes, Depressive' OR 'Unipolar Depression' OR 'Unipolar Depressions' OR 'unipolar disorder'):ab,ti,kw | 120588 |
| 7 | MeSH descriptor: [Animal Experimentation] explode all trees | 121 |
| 8 | MeSH descriptor: [Rats] explode all trees | 4073 |
| 9 | MeSH descriptor: [Rabbits] explode all trees | 1390 |
| 10 | ('Animal Experiment' OR 'Animal Experimental Use' OR 'Animal Experimental Uses' OR 'Animal Experimentation' OR 'Animal Experiments' OR 'animal physical conditioning' OR 'Animal Research' OR 'animal studies' OR 'animal study' OR 'animal trial' OR 'Belgian Hare' OR 'Chinchilla Rabbit' OR 'chronic unpredictable mild stress' OR 'CUMS' OR 'chronic restraint stress' OR 'CRS' OR 'Chinchilla Rabbits' OR 'cuniculus, Oryctolagus' OR 'Domestic Rabbit' OR 'Domestic Rabbits' OR 'domesticus, Mus musculus' OR 'Experiment, Animal' OR 'Experimental Use, Animal' OR 'Experimental Uses, Animal' OR 'Experimentation, Animal' OR 'Experiments, Animal' OR 'Hare, Belgian' OR 'House Mouse' OR 'House Mice' OR 'Laboratory Mouse' OR 'Laboratory Rat' OR 'Laboratory Mice' OR 'Laboratory Rats' OR 'leporid' OR 'Leporidae' OR 'leporids' OR 'Mice' OR 'Mice, House' OR 'Mice, Laboratory' OR 'Mice, Swiss' OR 'Mouse' OR 'Mouse, House' OR 'Mouse, Laboratory' OR 'Mouse, Swiss' OR 'Mus' OR 'Mus (genus)' OR 'Mus domesticus' OR 'Mus musculus' OR 'Mus musculus domesticus' OR 'New Zealand Rabbit' OR 'New Zealand White Rabbit' OR 'New Zealand White Rabbits' OR 'New Zealand Rabbits' OR 'newborn mice' OR 'norvegicus, Rattus' OR 'Norway Rat' OR 'Norway Rats' OR 'NZW Rabbit' OR 'NZW Rabbits' OR 'Oryctolagus cuniculus' OR 'physical conditioning, animal' OR 'Rabbit' OR 'Rabbit, Chinchilla' OR 'Rabbit, Domestic' OR 'Rabbit, New Zealand' OR 'Rabbit, NZW' OR 'Rabbits' OR 'rabbits and hares' OR 'Rabbits, Chinchilla' OR 'Rabbits, Domestic' OR 'Rabbits, New Zealand' OR 'Rabbits, NZW' OR 'Rat' OR 'Rat, Laboratory' OR 'Rat, Norway' OR 'Rats' OR 'Rats, Laboratory' OR 'Rats, Norway' OR 'Rattus' OR 'Rattus norvegicus' OR 'Research, Animal' OR 'Swiss Mouse' OR 'Swiss Mice' OR 'Zealand Rabbit, New' OR 'Zealand Rabbits, New'):ab,ti,kw | 32760 |
| 11 | (#1 OR #2 OR #3) AND (#4 OR #5 OR #6) AND (#7 OR #8 OR #9) | 158 |
|  |  |  |

**The search strategy (Wed of science)**

| Search number | Query | Results |
| --- | --- | --- |
| 1 | (TS=(Rabbits)) OR TS=(Rabbit or New Zealand Rabbits or Rabbit, New Zealand or Rabbits, New Zealand or Zealand Rabbit, New or Zealand Rabbits, New or New Zealand White Rabbit or New Zealand Rabbit or New Zealand White Rabbits or NZW Rabbits or NZW Rabbit or Rabbit, NZW or Rabbits, NZW or Rabbit, Domestic or Domestic Rabbit or Domestic Rabbits or Rabbits, Domestic or Oryctolagus cuniculus or cuniculus, Oryctolagus or Belgian Hare or Hare, Belgian or Chinchilla Rabbits or Chinchilla Rabbit or Rabbit, Chinchilla or Rabbits, Chinchill) | 1290 |
| 2 | (TS=(Mice)) OR AB=(Mus or Mouse or Mus domesticus or Mus musculus domesticus or domesticus, Mus musculus or Mus musculus or Mice, House or House Mice or Mouse, House or House Mouse or Mouse, Swiss or Swiss Mouse or Swiss Mice or Mice, Swiss or Mice, Laboratory or Laboratory Mice or Mouse, Laboratory or Laboratory Mouse) | 2889 |
| 3 | (TS=(Rats)) OR AB=(Rat or Rattus or Rattus norvegicus or Rats, Norway or Rats, Laboratory or Laboratory Rat or Laboratory Rats or Rat, Laboratory) | 5632 |
| 4 | (TS=(Animal Experimentation)) OR AB=(Experimentation, Animal or Animal Research or Research, Animal or Animal Experimental Use or Animal Experimental Uses or Experimental Use, Animal or Experimental Uses, Animal or Animal Experiments or Animal Experiment or Experiment, Animal or Experiments, Animal) | 189 |
| 5  6  7 | #1 OR #2 OR #3 OR #4  (TS=(Acupuncture Therapy)) OR AB=(Acupuncture Treatment or Acupuncture Treatments or Treatment, Acupuncture or Therapy, Acupuncture or Pharmacoacupuncture Treatment or Treatment, Pharmacoacupuncture or Pharmacoacupuncture Therapy or Therapy, Pharmacoacupuncture or Acupotomy or Acupotomies)  (TS=(Depressive Disorder)) OR AB=(Depressive Disorders or Disorder, Depressive or Disorders, Depressive or Neurosis, Depressive or Depressive Neuroses or Depressive Neurosis or Neuroses, Depressive or Depression, Endogenous or Depressions, Endogenous or Endogenous Depression or Endogenous Depressions or Depressive Syndrome or Depressive Syndromes or Syndrome, Depressive or Syndromes, Depressive or Depression, Neurotic or Depressions, Neurotic or Neurotic Depression or Neurotic Depressions or Melancholia or Melancholias or Unipolar Depression or Depression, Unipolar or Depressions, Unipolar or Unipolar Depressions) | 36784  28956  120368 |
| 8 | #5 AND #6 AND #7 | 326 |

Supplementary Table 2: Characteristics of included studies

| Studies | Animal models | | | Intervention(treatment group) | | | | Intervention(control group) | Cytokines and inflammatory mediators | Behavioral tests |
| --- | --- | --- | --- | --- | --- | --- | --- | --- | --- | --- |
|  | species | n=treatment group/control group | method of establishing AD | type | acupoints | treatment time and duration | electroacupuncture parameters |  |  |  |
| Guo T et al., 2014 | SD rat | 8/8 | CUMS | EA | GV20,GV29 | 20 minutes once everyday for 3 weeks | 2Hz, 1mA | Same conditions without any treatment | IL-1β, IL-6 | Open Field Test, Body Weight |
| Lu J et al., 2016 | SD rat | 8/8 | CUMS | MA | GV20,PC6 | 11 minutes each time, every other day for 4 weeks | / | Same conditions without any treatment | IL-1β, IL-6, TNF-α | Body Weight, Open field test |
| Yue N et al., 2018 | SD rat | 8/9 | CUMS | EA | GV20,GB34 | 30 minutes every other day for 4 weeks | 2Hz, 0.3mA | Same conditions without any treatment | IL-1β, IL-6, TNF-α |  |
| Cai W et al., 2019 | SD rat | 8/8 | CUMS | EA | GV20,DU24 | 30 minutes each time, 5 times a week for 4 weeks | 2Hz | Same conditions without any treatment | IL-1β, IL-6, TNF-α |  |
| Zhang K et al., 2020 | Wistar rat | 8/8 | CUMS | EA | GV20，GV29 | 30 minutes every day for 1 week | 2Hz, intermittent wave | Same conditions without any treatment | IL-1β, IL-6, TNF-α | Open field test |
| Jung J et al., 2021 | C57BL/6 mice | 8/7 | CRS | MA | KI10, LR8, LU8, LR4 | 30 minutes once every day for 1 week | / | Same conditions without any treatment | IL-1β, TNF-α | Open field test |
| Liao H et al., 2021 | C57BL/6 mice | 10/10 | CUMS | EA | ST36 | 20 minutes each time, 3 times a week, 2 weeks | 2Hz, 1mA,100 μs pulse width | Same conditions without any treatment | IL-1β, IL-4,, IL-6, IL-10, TNF-α |  |
| Chen L et al., 2022 | SD rat | 11/11 | CRS | MA | GV20,GV29 | 20 minutes once everyday for 3 weeks | / | Same conditions without any treatment | IL-10 | Body Weight |
| Chen Y et al., 2022 | SD rat | 9/9 | CUMS | MA | GV23,GV16 | 20 minutes, every other day for 4 weeks | / | Same conditions without any treatment | IL-1β, IL-6, TNF-α | Body weight, Open field test |
| Wang Q et al., 2022 | C57BL/6 mice | 10/10 | CUMS | EA | GV20, BL23,KI3 | 15 minutes once everyday for 3 weeks | 2 Hz, 2 V, 0.6mA,sparse wave | Same conditions without any treatment | IL-6, IL-1β，TNF-α |  |
| Chen W et al., 2023(1) | SD rat | 10/10 | CUMS | MA | GV16 GV23 | 20 minutes once every other day for 4 weeks | / | Same conditions without any treatment | IL-4 | Open field test, Body weight |
| Chen W et al., 2023(2) | SD rat | 9/9 | CUMS | MA | GV16 GV23 | 20 minutes every other day for 4 weeks | / | Same conditions without any treatment | IL-1β, IL-6, TNF-α |  |
| Han C et al., 2002 | SD rat | 13/13 | CUMS | EA | GV20，SP6 | 30 minutes once everyday for 3 weeks | 4.5V, 20Hz, sparse wave | Same conditions without any treatment | IL-1β, IL-6 | Open field test, Body weight |
| Li L et al., 2005 | Wistar rat | 14/14 | CUMS | EA | GV20、LR3、RN17、PC6 | 30 minutes once everyday for 3 weeks | 9V, 2Hz, sparse wave | Same conditions without any treatment | IL-6, TNF-α | Open field test, Body weight |
| Hu L et al., 2013 | SD rat | 8/8 | CUMS | MA | GV20、PC6 | 10 minutes every other day for 4 weeks | / | Same conditions without any treatment | IL-1β, IL-6, TNF-α | Open field test, Body weight |
| Yu M et al., 2016 | SD rat | 8/8 | CUMS | MA | GV20、GV29 | 30 minutes once everyday for 3 weeks | / | Same conditions without any treatment | IL-6, TNF-α | Open field test, Body weight |
| Jiang H et al., 2018 | SD rat | 11/11 | CUMS | MA | GV20、GV29 | 20 minutes once everyday for 3 weeks | / | Same conditions without any treatment | IL-1β,IL-10,TNF-α | Open field test, Body weight |
| Zhang R et al., 2018 | SD rat | 10/10 | CUMS | EA | GV20、HT7、LR3 | 30 minutes once everyday for 4 weeks | 2Hz, sparse wave | Same conditions without any treatment | IL-6 | Open field test, Body weight |
| Lv Z et al., 2020 | SD rat | 10/10 | CUMS | EA | GV20、HT7、LR3 | 20 minutes each time, 5 times a week for 4 weeks | 2mA, sparse wave | Same conditions without any treatment | IL-10, IL-1β | Body weight |
| Zhao Y et al., 2020 | SD rat | 8/8 | CUMS | MA | GV20、GV29 | 10 minutes each time, 6 times a week for 6 weeks | / | Same conditions without any treatment | IL-1β, IL-6, IL-4, IL-10 | Open field test |
| Li X et al., 2021 | SD rat | 8/8 | CUMS | MA | GV20、GV29 | 10 minutes each time, 6 weeks | / | Same conditions without any treatment | IL-1β, IL-6 | Open field test |
| Qi W et al., 2022 | ICR mice | 10/10 | CUMS | MA | GV20，HT7，SP6 | 10 minutes each time, 3 weeks | / | Same conditions without any treatment | TNF-α | Body weight |
| Li ,X,Y et al., 2021 | SD rat | 8/8 | CUMS | MA | GV20,GV29 | 30 minutes every day for 6 weeks |  | Same conditions without any treatment | IL-1β | Open field test |
| Tong T et al., 2024 | SD rat | 10/10 | CUMS | EA | GV23,GV16 | 20 minutes every day for 4 weeks | 0.5mA, 2Hz, intermittent wave | Same conditions without any treatment | IL-1β, TNF-α | Body weight |
| Zhou F et al., 2022 | SD rat | 8/8 | CUMS | EA | ST36,SP6 | 30 minutes every day for 2 weeks | 0.2mA, 5Hz | Same conditions without any treatment | IL-1β |  |

Supplementary table 3: Results from Egger’s test and trim and fill analysis

| Outcomes | Egger's test | Before trim and fill | | | After trim and fill | | |
| --- | --- | --- | --- | --- | --- | --- | --- |
|  | *P-value* | *P-value* | Est (F/R) | No. studies | *P-value* | Est (F/R) | No. studies |
| IL-1β | 0.008 | <0.001 | -1.620/-1.721 | 19 | <0.001 | -1.620/-1.721 | 19 |
| IL-6 | <0.001 | <0.001 | -1.629/-1.887 | 16 | <0.001 | -1.629/-1.887 | 16 |
| TNF-α | 0.008 | <0.001 | -1.829/-2.089 | 15 | <0.001 | -1.829/-2.089 | 15 |
| body weight | <0.001 | <0.001 | 1.559/1.688 | 14 | <0.001 | 1.324/1.367 | 17 |
| OFT | 0.011 | <0.001 | 1.642/1.772 | 11 | <0.001 | 1.353/1.398 | 14 |

Est, total effect sizes; F/R, fixed effect model/random-effects model; No., number.
